# Exploring the Antimicrobial and Antitumoral Activities of Naphthoquinone-Grafted Chitosans

**DOI:** 10.3390/polym15061430

**Published:** 2023-03-14

**Authors:** Fernanda Petzold Pauli, Cyntia Silva Freitas, Patricia Ribeiro Pereira, Alviclér Magalhães, Fernando de Carvalho da Silva, Vania M. F. Paschoalin, Vitor Francisco Ferreira

**Affiliations:** 1 Departamento de Tecnologia Farmacêutica, Faculdade de Farmácia, Universidade Federal Fluminense, Niterói 24241-000, Brazil; fernandapauli@id.uff.br; 2 Departamento de Bioquímica, Instituto de Química, Universidade Federal do Rio de Janeiro, Rio de Janeiro 21941-909, Brazil; freitas.cs@pos.iq.ufrj.br (C.S.F.); patriciarp@iq.ufrj.br (P.R.P.); 3 Departamento de Química Orgânica, Instituto de Química, Universidade Federal do Rio de Janeiro, Rio de Janeiro 21941-909, Brazil; alvicler@iq.ufrj.br; 4 Departamento de Química Orgânica, Instituto de Química, Universidade Federal Fluminense, Niterói 24020-141, Brazil; fcsilva@id.uff.br

**Keywords:** drug delivery, preclinical tests, antibacterial and anti-human mammary adenocarcinoma effects, chemotherapeutic adjuvant, in vitro toxicity evaluation, RAMAN, ssNMR

## Abstract

Biopolymers obtained from natural macromolecules are noteworthy among materials presenting high biocompatibility and adequate biodegradability, as is the case of chitosan (CS), making this biopolymeric compound a suitable drug delivery system. Herein, chemically-modified CS were synthetized using 2,3-dichloro-1,4-naphthoquinone (1,4-NQ) and the sodium salt of 1,2-naphthoquinone-4-sulfonic acid (1,2-NQ), producing 1,4-NQ-CS and 1,2-NQ-CS by three different methods, employing an ethanol and water mixture (EtOH:H_2_O), EtOH:H_2_O plus triethylamine and dimethylformamide. The highest substitution degree (SD) of 0.12 was achieved using water/ethanol and triethylamine as the base for 1,4-NQ-CS and 0.54 for 1,2-NQ-CS. All synthesized products were characterized by FTIR, elemental analysis, SEM, TGA, DSC, Raman, and solid-state NMR, confirming the CS modification with 1,4-NQ and 1,2-NQ. Chitosan grafting to 1,4-NQ displayed superior antimicrobial activities against *Staphylococcus aureus* and *Staphylococcus epidermidis* associated with improved cytotoxicity and efficacy, indicated by high therapeutic indices, ensuring safe application to human tissue. Although 1,4-NQ-CS inhibited the growth of human mammary adenocarcinoma cells (MDA-MB-231), it is accompanied by cytotoxicity and should be considered with caution. The findings reported herein emphasize that 1,4-NQ-grafted CS may be useful in protecting injured tissue against bacteria, commonly found in skin infections, until complete tissue recovery.

## 1. Introduction

A growing interest in the development of novel biomolecule-based non-cytotoxic and biocompatible pharmacological substances has been noted in recent years [1]. Biopolymers derived from natural macromolecules are noteworthy among materials presenting special properties due to their high biocompatibility and adequate biodegradability. One of the most employed biopolymers for this purpose is chitosan (CS), obtained by partial deacetylation of chitin, the second most abundant polymer in nature [2]. Structurally, chitin is an insoluble polysaccharide comprising repeated *N*-acetyl-D-glucosamine (GlcNAc) units bound by β-(1→4) glycosidic bond, whereas CS is a linear β-1,4-linked GlcNAc and D-glucosamine (GlcN) polysaccharide [2]. The relative GlcNAc to GlcN CS monomer ratio is determined by the deacetylation degree (DD), usually ranging from 60 to 95% [3].

As chitin presents limited solubility, restricted to non-conventional and toxic solvent systems, as well as limited thermal lability compared to CS, which is a lower molecular weight compound with better solubility and lower viscosity than chitin in water at any given pH [4], chitin applications are limited compared to CS [5]. Chitosan, in addition to being biocompatible, biodegradable, and non-toxic, also displays antimicrobial properties and can inhibit the growth of a variety of fungi and bacteria [6,7,8,9]. 

In this context, chemical modifications have emerged as a strategy to improve antimicrobial CS activity [10,11]. One of the most attractive structural modification methods consists in binding bioactive substances to CS through covalent bonds [12]. Three types of active groups present in the CS structure provide the opportunity for chemical modifications, namely the amino group and the primary and secondary hydroxyl groups located at the C-3 and C-6 positions. One of the proposed modifications in this regard is alkylation. In view of hydroxyl group ratios, the C-3 primary hydroxyl group presents less steric hindrance than the C-6 secondary hydroxyl group [13]. This conjugation can maintain fundamental CS properties and favor new biological properties attributed to small active molecules [14,15].

A class of bioactive substances of biological interest comprises naphthoquinones (NQS), that exist in two isomeric forms according to the carbonyl group position, i.e., 1,2- and 1,4-naphthoquinone. Despite carbonyl positions, both isomers present conjugated double bonds and a completely delocalized π-electron system [16]. 

One of the simplest exclusively synthetic naphthoquinones is 2,3-dichloro-1,4-naphthoquinone, largely employed in organic synthesis processes as a precursor for other heterocycles and naphthoquinone derivatives [17]. This quinone and its derivatives are also noteworthy due to their biological properties, such as antibacterial, antifungal, trypanocide and antimalarial activities [18,19,20,21]. Moreover, 2,3-dichloro-1,4-naphthoquinone is also known as Dichlone, a commercial fungicide [17].

Another synthetic naphthoquinone widely applied in organic synthesis processes is 1,2-naphthoquinone-4-sulfonic acid [22]. This quinone is well known for its application in analytical derivatization techniques, mainly as a colorimetric indicator named Follin’s reagent, routinely used to identify and qualify amines and amino acids by the formation of colored products [22]. Many different uses of this functionalization employing this naphthoquinone are available, producing derivatives with diverse biological activity, such as antitumor and anti-inflammatory activities [23,24,25].

The significant advantage of using naphthoquinones and their derivatives as new antimicrobial candidates is the fact that their redox cycle produces reactive oxygen (ROS) and nitrogen (RNS) species that are harmful towards microorganisms, damaging intracellular structures and leading to the disassembly of plasmatic microorganism membranes [26,27]. 

Despite the rising interest and increasing number of publications concerning chemically modified CS, to the best of our knowledge, no studies on the characterization of CS structurally modified through covalent bonding with 1,2-naphthoquinone-4-sulfonic acid or 2,3-dichloro-1,4-naphthoquinone are available. Thus, the aim of the present investigation was to synthesize, characterize and investigate the antibacterial and antitumoral activities of chemically modified CS grafted with the sodium salts of 1,2-naphthoquinone-4-sulfonic acid or 2,3-dichloro-1,4-naphthoquinone.

## 2. Material and Methods

### 2.1. Experimental Design

**Figure 1:**
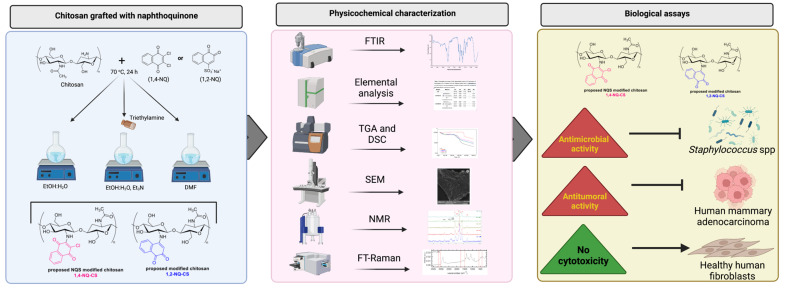
Experimental steps and analysis of naphthoquinone−grafted chitosans. Production of chitosan-naphthoquinone derivatives were performed by three different methods followed by analyses and physicochemical characterizations of the modified polymers, evaluation of antimicrobial and antitumoral effectiveness and cytotoxicity tests.

Chitosan was grafted with the naphthoquinones 1,2-naphthoquinone-4-sodium-sulfonate (1,2-NQ) or 2,3-dicoloro-1,4-naphthoquinone (1,4-NQ) by three different methods, using ethanol and water (EtOH:H2O), ethanol, water and triethylamine (EtOH:H2O, Et2N) or only DMF, generating six formulations, as it can be seen in Figure 1. After preparing the grafted-chitosans, physicochemical characterization analysis were carried out to investigate and confirm the modification of the polymer throughs, using FTIR, elemental analysis, TGA, and DSC, SEM, NMR and FT-Raman. Grafted-chitosans were screened by biological tests in order to assess their antimicrobial and antitumoral efficacies, followed by cytotoxicity tests with healthy tohuman healthy cells, in order to evaluate their biocompatibility in preserv-ing the surrounding healthy tissues (Figure 1).

### 2.2. Materials

Low molecular weight CS (50,000–190,000 Da), ethyl alcohol, dimethyl formamide, triethylamine, sodium 1,2-naphthoquinone-4-sulfonate (1,2-NQ) and 2,3-dicholoro-1,4-naphthoquinone (1,4-NQ) were purchased from Sigma Aldrich Co., Rockville, MD, USA.

### 2.3. Preparation of the Modified Chitosan Naphthoquinone Derivatives

Chitosans grafted with sodium 1,2-naphthoquinone-4-sulfonate (1,2-NQ-CS) were prepared employing three different synthetic methodologies using a fixed amount of 160 mg chitosan and 1 mmol (260 mg) of sodium 1,2-naphthoquinone-4-sulfonate (1,2-NQ). 

Method a—EtOH:H_2_O: sodium 1,2-naphthoquinone-4-sulfonate was transferred to a 100 mL round-bottom flask containing a 20 mL ethanol/water mixture (1:1). After stirring at 70 °C for 24 h, the mixture was filtered and washed with ethanol, hexane, and anhydrous acetone. Next, 1,2-NQ-CS was dried for 3 h at 60 °C in an oven, following storage in a desiccator under vacuum at room temperature. 

Method b—EtOH:H_2_O plus triethylamine (Et_3_N): CS and sodium 1,2-naphthoquinone-4-sulfonate, at the same ratio as described above, were mixed with 0.24 mL of triethylamine in a 100 mL round-bottom flask containing a 20 mL ethanol/water mixture (1:1). The mixture was stirred at 70 °C for 24 h and the 1,2-NQ-CS products were isolated as described in method a. 

Method c—dimethylformamide (DMF): chitosan and sodium 1,2-naphthoquinone-4-sulfonate were mixed in a 100 mL round-bottom flask at the same ratio as described above containing 20 mL of DMF. The mixture was stirred at 70 °C for 24 h, and the 1,2-NQ-CS products were isolated as described in method a. 

The products from chitosan grafted with 2,3-dicholoro-1,4-naphtoquinone (1,4-NQ-CS) were also synthesized using the same three methods as described above, albeit employing 1 mmol (227 mg) of 2,3-dicholoro-1,4-naphtoquinone instead of 1,2-NQ.

### 2.4. Physicochemical Characterizations

The FTIR analyses were performed using a Thermo Nicolet iS-50 spectrometer (Thermo Fisher, Waltham, MA, USA) equipped with an Attenuated Total Reflectance (ATR) module from 4000 to 450 cm^−1^ during 32 scans at 4 cm^−1^ resolution.

Thermogravimetric curves were obtained using a Shimadzu TGA-60 instrument (Shimadzu, Columbia, MD, USA) under an inert nitrogen atmosphere at a 50 mL/min flow rate and 10 °C·min^−1^ heating rate, from 30 °C to 500 °C, and initial mass of about 5 mg of solid sample. 

Differential scanning calorimetry (DSC) was performed using a Shimadzu DSC-60 instrument (Shimadzu, Columbia, MD, USA) from 25 °C to 500 °C at a 10 °C·min^−1^ heating rate under a nitrogen atmosphere. 

The elemental analyses were performed using a Perkin Elmer 2400 series II (Perkin Elmer, Waltham, MA, USA). According to Rahman et al., 2019 [28], the results may be used to determine the degree of substitution (DS), according to Equation (1):

(1) $$DS=\frac{{\left(\frac{C}{N}\right)}_{mc}-{\left(\frac{C}{N}\right)}_{c}}{n}$$

where ${\left(\frac{C}{N}\right)}_{mc}$ corresponds to the modified CS (*mc*) Carbon/Nitrogen (ratio $\left(\frac{C}{N}\right)$, ${\left(\frac{C}{N}\right)}_{c}$ is the C/N of pure CS (*c*) and *n* is the number of extra carbon atoms introduced from the quinone. The elemental analysis technique also allows for the determination of the CS deacetylation degree (DD), according to Equation (2) [29]:

(2) $$DD=\left(1-\frac{{\left(\frac{C}{N}\right)}_{c}-5.145}{6.861-5.145}\right)\times 100$$

where 5.145 corresponds to the *N*-deacetylated repeat unit (C_6_H_11_O_4_N) and 6.861, to the fully N-acetylated repeat unit (C_8_H_13_O_5_N).

Scanning electron microscopy (SEM) analyses were employed to study CS surface and morphology. Samples were deposited on stubs containing carbon tape and metallized with gold and visualized using a SEM JEOL JSM-6460LV equipment (JEOL, San Jose, CA, USA). Images were taken by applying an electron beam accelerating voltage of 15 kV.

^13^C solid-state NMR was performed at 125 MHz using Bruker NMR 11.7T Avance Neo spectrometer operating at 500 MHz, equipped with a PH MAS VTN (N-P, F-H) 4 mm probe head. Around 1024 scans were acquired using a 4 mm zirconium rotor under MAS at 10 KHz and cross-polarization (CP) applying 5 ms of contact time.

The FT-Raman spectra were obtained using a Bruker MultiRAM spectrometer (Bruker, Billerica, MA, USA) between 3600 and 100 cm^−1^ with a 2 cm^−1^ resolution using Nd:YAG laser line as the excitation source (1064 nm). The samples were measured in the hemispheric opening of an aluminum sample holder, and the spectra were recorded at room temperature with a germanium detector maintained in liquid nitrogen.

### 2.5. Biological Assays

#### 2.5.1. Organisms and Human Cell Lineages

The clinically relevant microorganisms *Staphylococcus aureus* (ATCC 14458) and *Staphylococcus epidermidis* (ATCC 12228) were used in the antimicrobial susceptibility tests, kindly provided by the FIOCRUZ-INCQS cell bank.

A healthy human fibroblast cell lineage, HFF-1 (ATCC SCRC-1041), and breast adenocarcinoma cell lineage, MDA-MB-231 ATCC ^®^ HTB-26™), were purchased from the Rio de Janeiro Cell Bank (BCRJ) and used for toxicological tests and antitumoral activity assays performed in vitro through cell viability assays following antitumoral cell exposure to the naphthoquinone-grafted CS.

#### 2.5.2. Antimicrobial Activity Assessments of Naphthoquinones, Chitosan and Chitosan-Grafted with Naphthoquinones

Antimicrobial activities were tested against pathogenic bacteria through the microdilution method to estimate the minimum inhibitory concentration (MIC) according to Clinical and Laboratory Standards Institute (CLSI) recommendations, with adaptations [30].

Bacteria were inoculated in Mueller Hinton Broth (MHB) containing 2.0 g/L of meat extract, 17.5 g/L of casamino acid and 1.5 g/L of starch (KASVI, PR, BR) and incubated at 37 °C for 18 h under constant agitation in aerobic conditions. Subsequently, a bacterial suspension containing 10^8^ cells prepared according to the McFarland 0.5 scale was 10-fold serial diluted in MHB. Aliquots of the free naphthoquinones, CS and naphthoquinone-grafted CS were 2-fold serially diluted, beginning at 5 mg/mL, and added to the bacterial suspension at a final concentration of 10^7^ cells/mL. The microplates were then incubated at 37 °C for 18 h under constant agitation and cell viability was assessed by adding 30 µL of 0.02% resazurin, followed by a further incubation at 37 °C for 2 h according to McMillian et al. [31]. Fluorescence intensities were determined using a 2030 Multilabel Reader VICTOR™ X4 microplate reader (Perkin Elmer, Waltham, MA, USA) at 530 nm (excitation) and 590 nm (emission).

#### 2.5.3. Cytotoxicity Evaluations of Free-Naphthoquinones, Chitosan and Chitosan Grafted with Naphthoquinones towards Healthy Human Cell Lineages

Toxicological screening of the free-naphthoquinones, CS and grafted CS against the healthy human fibroblast cell lineage HFF-1 (ATCC SCRC-1041) was performed on cell cultures at a concentration of 5 × 10^5^ cells/mL plated in 96-well microplates in Dulbecco’s modified Eagle’s high glucose medium (DMEM, Ref# 11330-032) (Gibco, Billings, MT, USA) supplemented with 15% fetal bovine serum (FCS) [32]. To allow cell adhesion, the microplates were incubated at 37 °C for 24 h under a humidified atmosphere containing 5% CO_2_. The samples were 2-fold serially diluted, starting at 2.5 mg/mL and then added to a semi-confluent cell monolayer, followed by incubation for a further 24 h. Cell viability was assessed by adding 20 µL aliquot of 125 µg/mL resazurin to each well (Sigma Aldrich Co., St. Louis, MO, USA), according to McMillian et al. [31], and fluorescence intensities were determined after 4 h of incubation using a Victor™ X microplate reader (Perkin Elmer, Waltham, MA, USA) at excitation and emission wavelengths of 530 and 590 nm.

The GraphPad Prism software (version 7, GraphPad, San Diego, CA, USA) was used to construct the healthy and tumoral cell growth inhibition plots for each sample tested in a log scale, in order to determine the CC_50_, and IC_50_, respectively, for free-naphthoquinones, CS and naphthoquinone-grafted CS. The CC_50_/IC_50_ ratios, defined as the selective index, were then calculated.

#### 2.5.4. Assessment of In Vitro Antitumoral Activity of Free-Naphthoquinones, Chitosan and Chitosan Grafted with Naphthoquinones

The antitumoral activities of the naphthoquinones, CS and naphthoquinone-grafted CS were determined in vitro using the human breast adenocarcinoma cancer cell line, MDA-MB-231 (ATCC^®^ HTB-26™) by the resazurin redox method [32]. Tumoral cells (5 × 10^5^ cells/well) were seeded in 96-well microplates in Dulbecco’s modified Eagle medium (F-12 Nutrient Mix—DMEM/F-12) (Sigma-Aldrich Co., St. Louis, MO, USA), supplemented with 10% fetal calf serum (FCS), 2 mM L-glutamine, 5 × 10^5^ M 2-mercapto-ethanol. The plates were then incubated for 24 h at 37 °C under a humidified atmosphere containing 5% CO_2_ until a semiconfluent monolayer was achieved. Samples were serially diluted 2-fold from an initial concentration of 2.5 mg/mL and added to the semiconfluent cell monolayer followed by incubation for a further 24 h. The cytotoxicity analyses were performed similarly to the described above for the in vitro cytotoxicity test using the HFF-1 cell lineage. The GraphPad Prism software (version 7, GraphPad, San Diego, CA, USA) was used to plot the healthy and tumoral cells growth inhibition curves for each sample in a log scale, in order to determine the respective IC_50_ for free-naphthoquinones, CS and naphthoquinone-grafted CS. 

The CC_50_/IC_50_ ratios, defined as the selective index, were then calculated.

## 3. Results and Discussion

### 3.1. Synthesis

**Figure 2:**
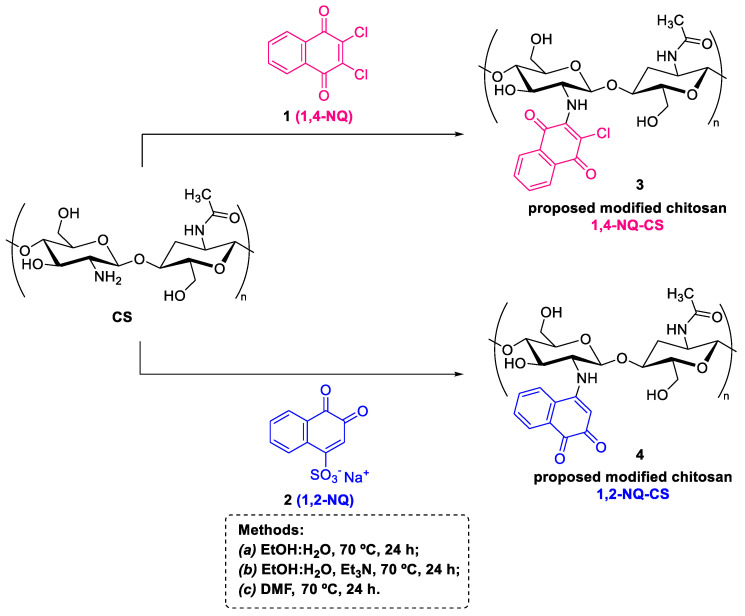
Synthesis of naphthoquinone modified CS using three different methodologies, namely an alcohol/water (EtOH:H_2_O) mixture, alcohol/water (EtOH:H_2_O) plus triethylamine, (Et_3_N) and alcohol/water (EtOH:H_2_O) dimethylformamide (DMF). The reaction took place at 70 °C for 24 h and the mixture was filtered and washed with ethanol, hexane, and anhydrous acetone.

The naphthoquinone-grafted CS were prepared employing three different reaction conditions to evaluate final polymer formation. Inspired by Freitas (2019) [33], the first approach was evaluated using ethanol and water as solvent (EtOH:H_2_O method), whereas the second method was inspired by Greco et al. (2015) [34], employing triethylamine as base (EtOH:H_2_O + Et_3_N method). In order to improve CS solubility, a third approach was tested by using dimethylformamide as solvent (DMF method). The proposed modified CS products are displayed in Figure 2. 

Three types of active chemical groups that provide opportunities for chemical modification are present in the chemical CS structure, namely the amino group, and the primary and secondary hydroxyl groups at the C-3 and C-6 positions [35]. The primary hydroxyl group C-3 presents less steric hindrance than the secondary hydroxyl group at C-6, although the amino group is considered more reactive than both hydroxyl groups [36]. In addition to steric hindrance, the greater nucleophilicity associated with the nitrogen atom compared to oxygen promotes a greater probability of an *N*-substitution product than an *O*-substitution product [6]. In this context, the Michael addition product 1,4-NQ-CS was proposed for the reaction between 2,3-dichloronaphthoquinone and CS, whereas the amino-1,2-naphthoquinone (1,2-NQ-CS) was proposed for the reaction between sodium 1,2-naphthoquinone-4-sulfonate and CS, based on similar reactions reported in the literature [20,37,38,39,40,41].

### 3.2. Infrared (IR) Spectroscopy

**Figure 3:**
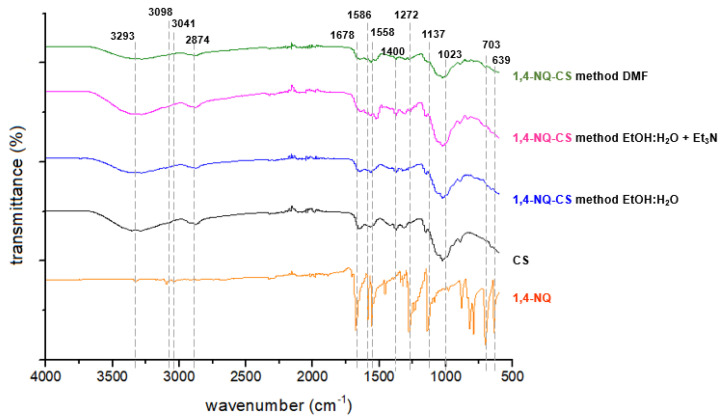
FTIR spectra of 2,3-dichloro-1,4-naphthoquinone (1,4-NQ), chitosan (CS), 1,4-NQ-CS obtained by the EtOH:H_2_O method, 1,4-NQ-CS following the EtOH:H_2_O + Et_3_N region and 1,4-NQ-CS following the DMF method.

**Figure 4:**
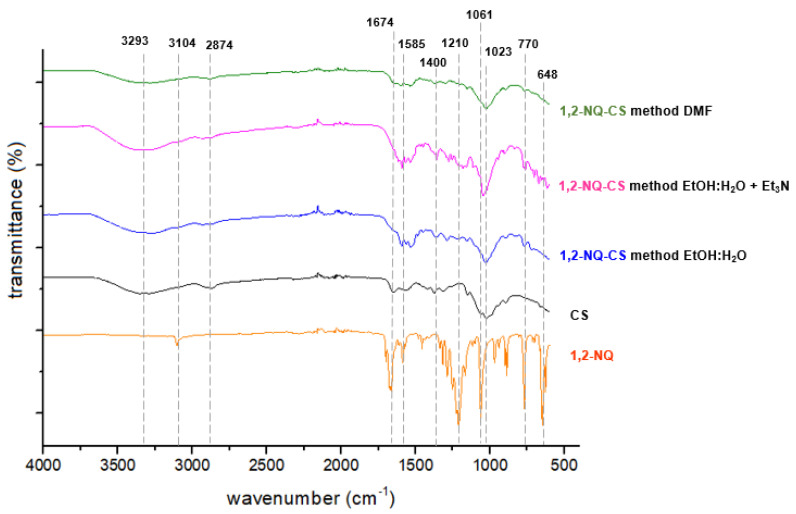
FTIR spectra of sodium 1,2-naphthoquinone-4-sulfonate (1,2-NQ), chitosan (CS), 1,2-NQ-CS obtained by the EtOH:H_2_O method, 1,2-NQ-CS following the EtOH:H_2_O + Et_3_N method and 1,2-NQ-CS following the DMF method.

The IR spectra of pure CS displayed absorption bands characteristic of its polysaccharide matrix (Figure 3 and Figure 4). The large absorption band at approximately 3293 cm^−1^ is due to the stretching of the hydroxyl and amine groups (OH and NH_2_) [42]. The bands at 2874 cm^−1^ and 1650 cm^−1^ correspond to the symmetric and asymmetric stretching of the C-H bond of alkyl groups and C=O bond of amide, respectively [43]. The bands at 1500, 1400 and 1050 cm^−1^ are associated with the absorption bending vibration of the amino group (NH_2_), bending vibration of the C-H bond and vibrations associated to the C-O bond present in the polymeric matrix, respectively [44].

The IR spectra of 1,4-NQ (1, Figure 3) and 1,2-NQ (2, Figure 4) exhibit characteristic absorption bands related to carbonyl stretching at 1678 cm^−1^ and 1674 cm^−1^ for each, respectively. All NQs exhibited an intense band in the 3100–3090 cm^−1^ region, attributed to C-H stretching and in the 1270–1290 cm^−1^ region, referring to C-O stretching. In addition, 1,4-NQ presented an intense absorption band at 703 cm^−1^, which can be attributed to the stretching of the C-Cl bond, whereas 1,2-NQ presented strong bands at 1210 and 1061 cm^−1^, due to the stretching of S=O and S-O bonds characteristic of the sulfonate group. Following the chemical modification reaction, all NQ-CS complexes displayed absorption bands similar to those observed for the polymeric CS matrix. The absence of S=O and S-O stretching bands in the 1,2-NQ-CS spectra strongly indicates a chitosan reaction with 1,2-NQ, as proposed in Figure 2. However, a predominance of CS bands was observed for 1,4-NQ-CS. For better band discrimination, the detailed spectra were included in the supplementary file (Figures S1–S9).

### 3.3. Elemental Analysis

The deacetylation degree (DD) of pure CS and the degree of substitution (DS) of the modified CS products were evaluated using the C% and N% values obtained by the elemental analyses (Table 1). The 1,2-NQ-CS product presented higher DS values than 1,4-NQ-CS, probably due to the easy and favorable reaction between 1,2-naphthoquinone-4-sulfonate and the CS amino group. Furthermore, the reaction method using triethylamine as base resulted in higher DS values independently of the naphthoquinone. Chitosan’s limited solubility in water is widely known and does not prevent its extensive use as a pharmaceutical or biomedical device. Herein, the degree of substitution of the conjugated chitosan was low, ranging from 0.06 to 0.54. Thus, the DS should have a minor impact on final product solubility (Table 1).

### 3.4. Thermogravimetric Analysis

**Figure 5:**
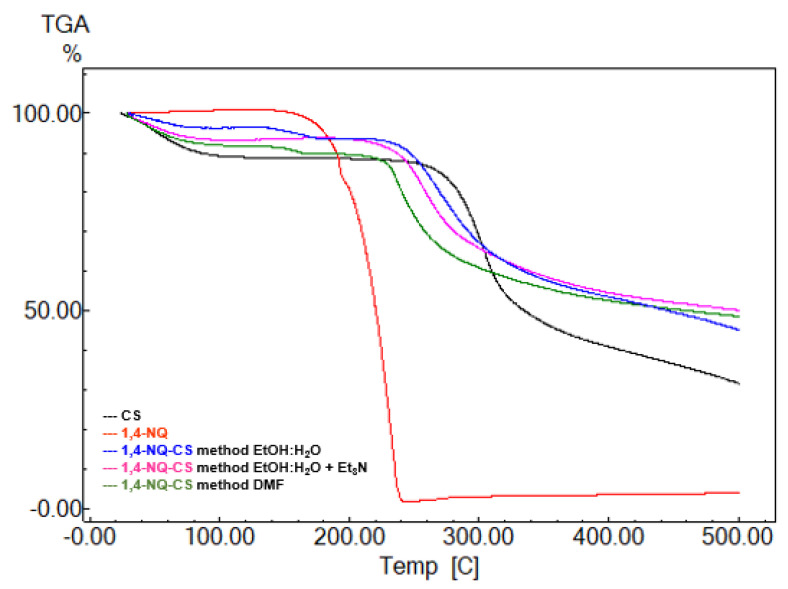
TGA thermograms of 2,3-dichloro-1,4-naphthoquinone (1,4-NQ), chitosan (CS), 1,4-NQ-CS obtained by the EtOH:H_2_O method, 1,4-NQ-CS following the EtOH:H_2_O + Et_3_N method and 1,4-NQ-CS following the DMF method.

**Figure 6:**
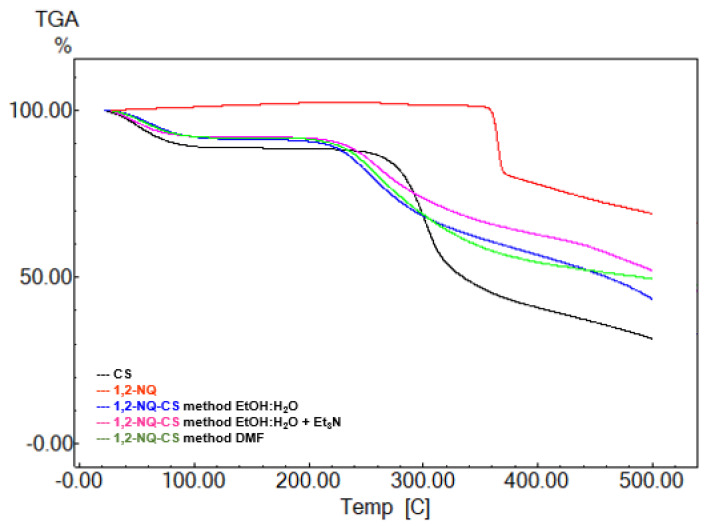
TGA thermograms of sodium 1,2-naphthoquinone-4-sulfonate (1,2-NQ), chitosan (CS), 1,2-NQ-CS obtained by the EtOH:H_2_O method, 1,4-NQ-CS following the EtOH:H_2_O + Et_3_N method and 1,4-NQ-CS following the DMF method.

A TGA analysis was performed to investigate the thermal and degradation properties of the modified CS. Figure 5 exhibits the TGA thermograms of CS, 1,4-NQ and its derivatives products, and Figure 6 displays the TGA thermograms of CS, 1,2-NQ and its derivatives. Pure CS displays two weight loss stages, the first related to the evaporation of physically absorbed water, with a 7% loss, and the second from 251 to 321 °C, attributed to polymer degradation, with a 38% loss [45]. According to the thermograms, all synthesized 1,4-NQ-CS and 1,2-NQ-CS exhibited an educed thermal decomposition temperature, probably due to CS crystallinity disruption due to chemical naphthoquinone introduction, promoting the loss of hydrogen bonds (Table 2) [46,47].

### 3.5. DSC Analysis

**Figure 7:**
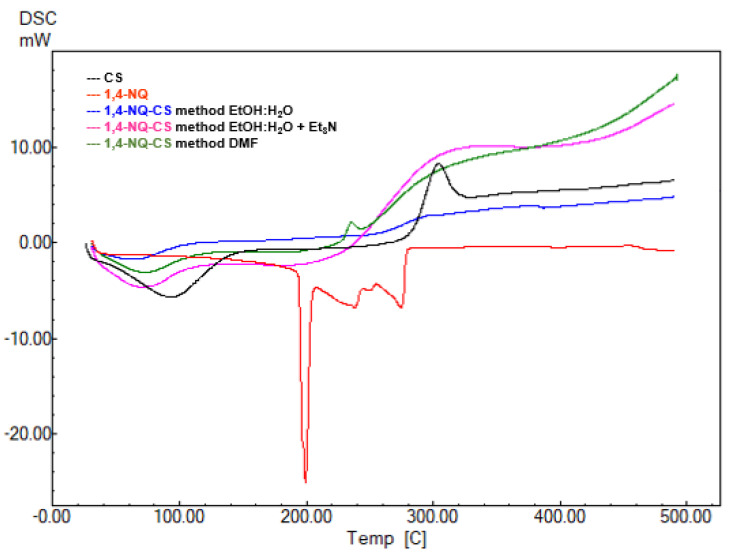
DSC thermograms of 2,3-dichloro-1,4-naphthoquinone (1,4-NQ), chitosan (CS), 1,4-NQ-CS obtained by the EtOH:H_2_O method, 1,4-NQ-CS following the EtOH:H_2_O + Et_3_N method and 1,4-NQ-CS following the DMF method.

**Figure 8:**
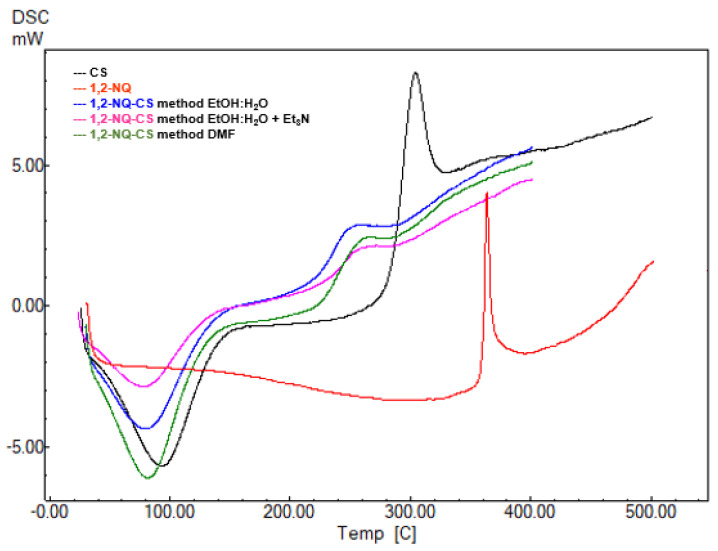
DSC thermograms of sodium 1,2-naphthoquinone-4-sulfonate (1,2-NQ), chitosan (CS), 1,2-NQ-CS obtained by the EtOH:H_2_O method, 1,2-NQ-CS following the EtOH:H_2_O + Et_3_N method and 1,2-NQ-CS following the DMF method.

The differential calorimetric thermograms of pure CS and chemically modified naphthoquinone 1,4-NQ-CS (Figure 7) and 1,2-NQ-CS are depicted in Figure 8. Pure CS displayed an endothermic peak at 93 °C and an exothermic peak around 304 °C corresponding to water loss and polymeric degradation [48]. After the reactions took place, the products 1,4-NQ-CS and 1,2-NQ-CS displayed decreases at the corresponding temperatures of both events, indicating altered CS structure by chemical NQ modifications, corroborating the TGA analysis (Table 2). 

### 3.6. SEM Analysis

**Figure 9:**
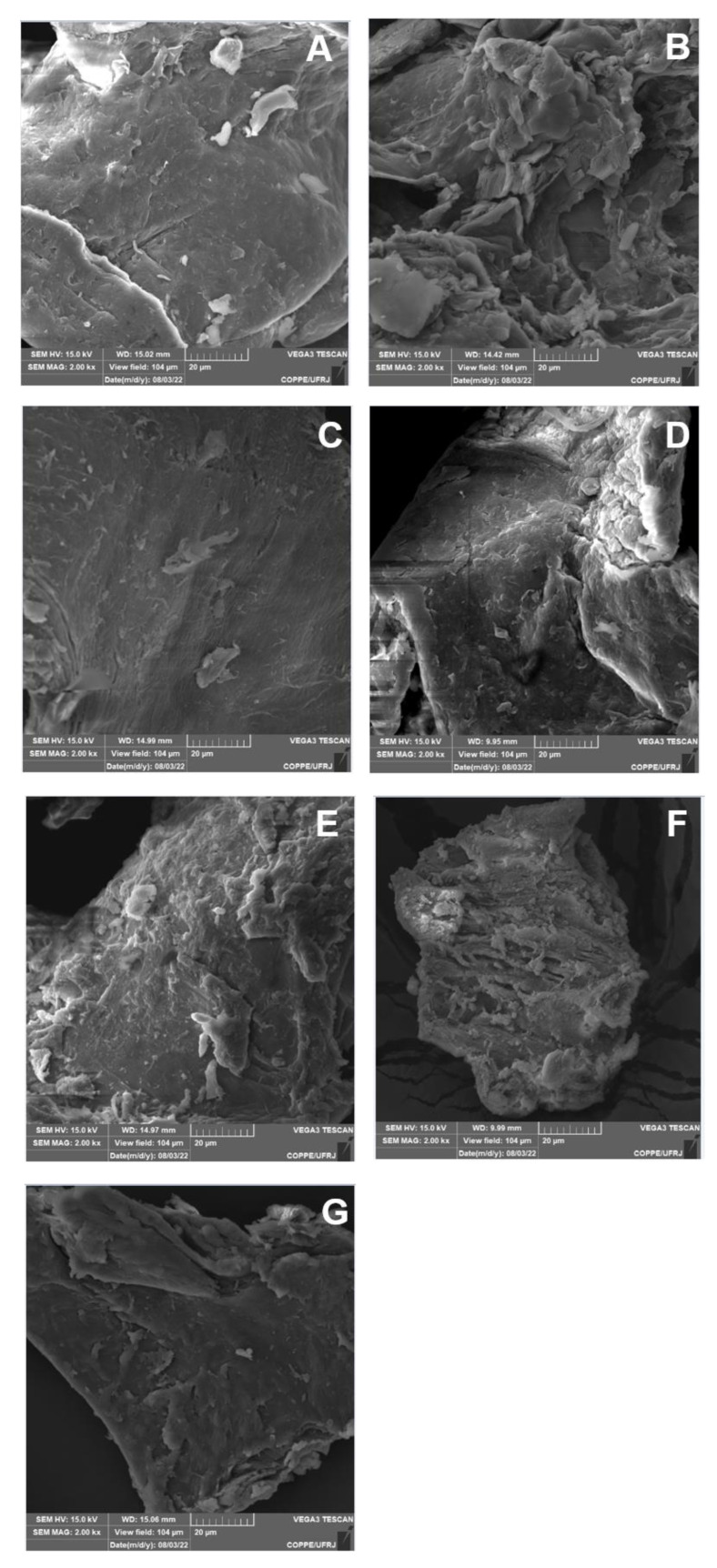
Morphological characterization of chitosan (**A**), 1,4-NQ-CS obtained following the EtOH:H_2_O method (**B**), 1,4-NQ-CS following the EtOH:H_2_O + Et_3_N method (**C**), 1,4-NQ-CS following the DMF method (**D**), 1,2-NQ-CS following the EtOH:H_2_O method (**E**), 1,2-NQ-CS following the EtOH:H_2_O + Et_3_N method (**F**), 1,2-NQ-CS following the DMF method (**G**), the structural ultraphotographies were acquired at 15 kV and 2000× magnification.

The scanning electron microscopy analysis of the chemically-modified CS displayed in Figure 9 presents the morphological structure of CS and the chemically-modified 1,4-NQ-CS (EtOH:H_2_O method), 1,4-NC-CS (EtOH:H_2_O + Et_3_N method), 1,4-NQ-CS (DMF method), 1,2-NQ-CS (EtOH:H_2_O method), 1,2-NQ-CS (EtOH:H_2_O + Et_3_N method) and 1,2-NQ-CS (DMF method). The surface roughness and pores of the modified CS were higher than that of CS itself. This was most significant in 1,2-NQ-CS, which presented the highest chemical modification degree, as DS values ranged from 0.39 to 0.54 when compared with 1,4-NQ-CS, which presented DS values ranging from 0.06 to 0.12 (Table 1). Polymeric chain interruption may explain this due to CS amino group naphthoquinone coupling.

### 3.7. Nuclear Magnetic Resonance (NMR) Spectroscopy

**Figure 10:**
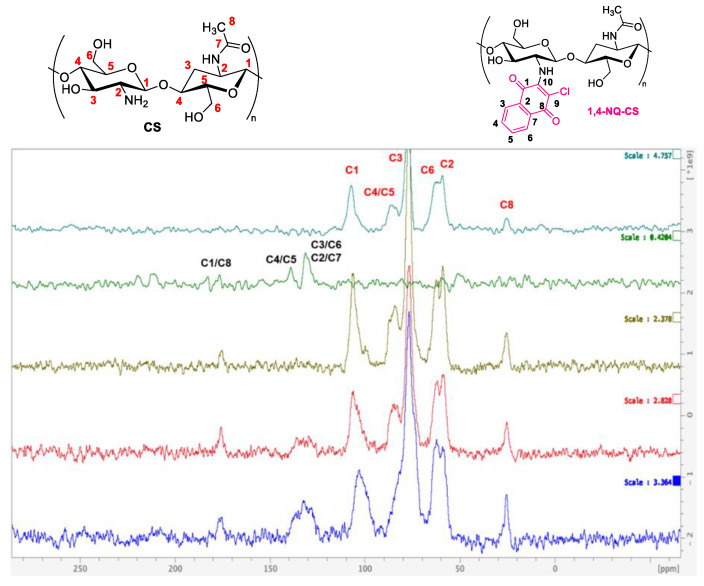
^13^C Solid State CP/MAS-10KHz NMR (125 MHz) spectra of chitosan (light green), 1,4-NQ (green), 1,4-NQ-CS obtained by method a (brown), 1,4-NQ-CS obtained by method b (red) and 1,4-NQ-CS obtained by method c (blue).

**Figure 11:**
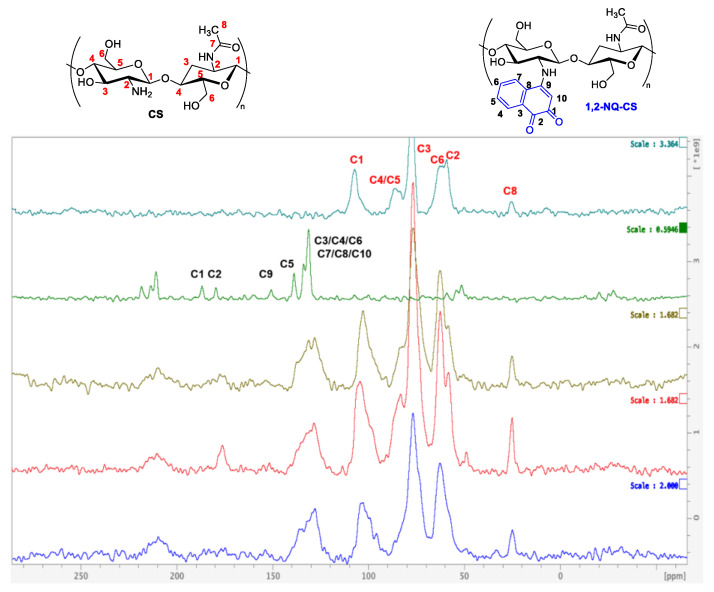
^13^C Solid State CP/MAS-10KHz NMR (125 MHz) spectra of chitosan (light green), 1,2-NQ (green), 1,2-NQ-CS obtained by method a (brown), 1,2-NQ-CS obtained by method b (red) and 1,2-NQ-CS obtained by method c (blue).

The NMR spectra of pure chitosan depicted in Figure 10 and Figure 11 present the characteristic peaks of the biopolymer moiety at 107, 86, 77, 62,59 and 23 ppm, assigned to C1, C4/C5, C3, C6, C2 and C8, respectively [49]. The pure 1,4-NQ spectra indicates signals at 177 (C1/C8 carbonyl), 139 (C4 and C5), 131 (C3 and C6) and 130 ppm (C2 and C7) (Figure 10). Compared to the CS spectra, new peaks are observed for the 1,4-NQ-CS products and attributed to the insertion of the 1,4-naphthoquinone moiety (Figure 10). Meanwhile, the pure 1,2-NQ spectra (Figure 11) presented peaks at 188 (C1), 180 (C2), 150 (C9), 139 (C5), 134 (C6 and C8), 131 ppm (C3, C4, C7 and C10). As expected, compared to CS, all 1,2-NQ-CS spectra exhibited new peaks attributed to 1,2-NQ (Figure 11). Therefore, for both NQ-CS cases, the peaks at 180 and 130 ppm reveal that the naphthoquinones were grafted to chitosan independently of the applied synthetic method. Detailed spectra are included in the supplementary file (Figures S10–S18).

### 3.8. Raman Spectroscopy

**Figure 12:**
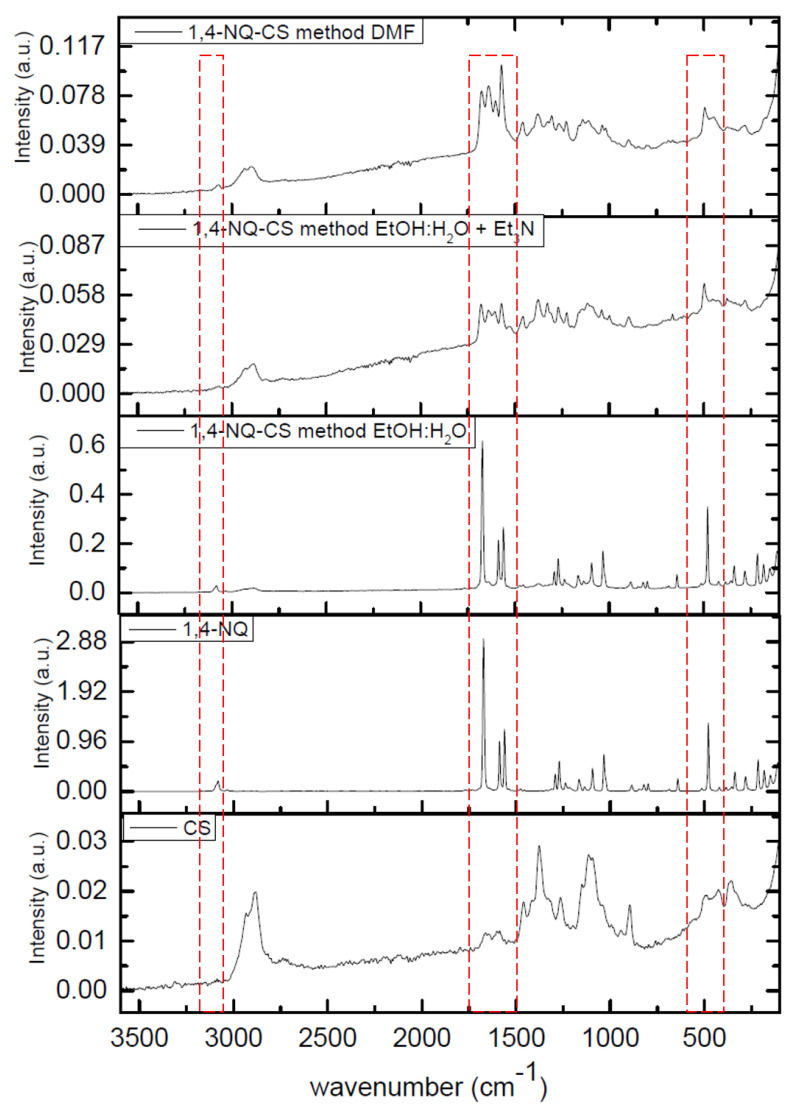
Experimental RAMAN spectra of 1,4-NQ-CS obtained by the DMF method; chitosan (CS); 1,4-NQ-CS obtained by the EtOH:H_2_O + Et_3_N method; 1,4-NQ-CS obtained by the EtOH:H_2_O method; 2,3-dichloro-1,4-naphthoquinone (1,4-NQ), and chitosan (CS).

**Figure 13:**
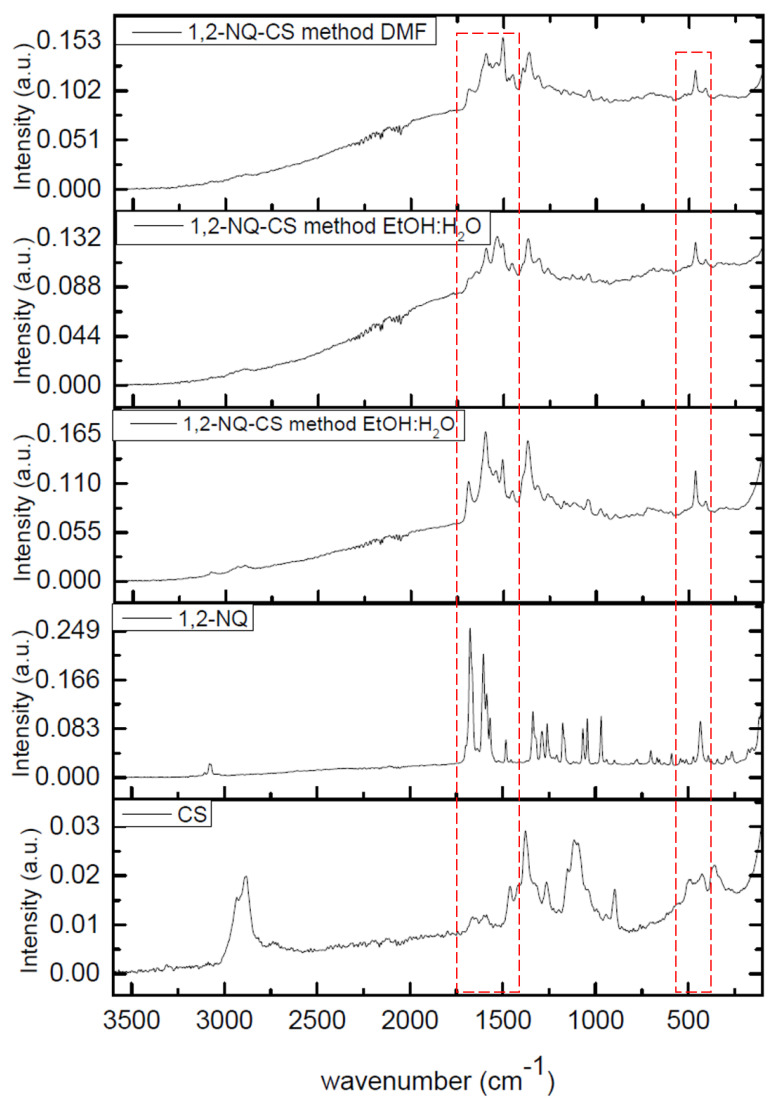
Raman spectra of 1,2-NQ-CS obtained by the 1,2-NQ-CS DMF method; 1,2-NQ-CS obtained by the EtOH:H_2_O method, 1,2-NQ-CS following the EtOH:H_2_O + Et_3_N method; sodium 1,2-naphthoquinone-4-sulfonate (1,2-NQ), and chitosan (CS).

The Raman spectra for chitosan are displayed in Figure 12 and Figure 13 and the main vibrational modes were assigned based on previous studies [50,51]. The Raman spectra of CS displayed a strong band at 2882 cm^−1^, assigned as the C-H aliphatic mode. The intense bands at 1380 cm^−1^ and 1110 cm^−1^ were assigned as δ(CH3) in the amide mode and ν(C−O−C) glucosyl ring mode.

The 3080 cm^−1^ band in the 1,4-NQ Raman spectra is characteristic of symmetrical aromatic C-H stretching of the naphthoquinone nucleus. The band at 1680 cm^−1^ was assigned as ν(C=O), while the bands at 11,561 cm^−1^ and 1586 cm^−1^ were assigned as ν(CC) aromatic modes from the quinone moiety. The 480 cm^−1^ band corresponds to the ν(C=CO) mode (Figure 12). A similar pattern was observed for 1,2-NQ (Figure 13), in which the 3070 cm^−1^ band was assigned as ν(CH aromatic). The stretching modes from the carbonyl group were assigned at 1680 cm^−1^, and the νCC aromatic modes of the quinone moiety were assigned at 1603 cm^−1^ and 1588 cm^−1^. The 433 cm^−1^ band refers to the quinone nucleus angle deformation ν(C=CO). The 1,4-NQ and 1,2-NQ spectra are similar to those from naphthoquinones reported in the literature [52,53].

The 1,4-NQ-CS spectra of the three samples indicated both chitosan and naphthoquinone bands (Figure 12). Similarly, to 1,2-NQ-CS, the presence of the assigned 1,2-NQ bands and chitosan bands were also noted (Figure 13). Therefore, bands corresponding to naphthoquinones were observed in both the 1,4-NQ-CS and 1,2-NQ-CS spectra, clearly indicating structural chitosan modifications.

### 3.9. Antibacterial Potential of Chitosan Grafted with Naphthoquinones

To evaluate antimicrobial activity effects of CS naphthoquinone grafting, two *Staphylococcus* species, *S. epidermidis* and *S. aureus*, considered of clinical and food interest, were grown in the presence of grafted or non-grafted CS or free naphthoquinones.

All compounds in their free forms, CS, 1,4-NQ and 1,2-NQ caused the complete inactivation of *S. aureus and S. epidermidis* growth at different concentrations and dependent on bacteria species (Table 3). The MIC values for *S. aureus* were 0.323, 0.625 and 0.156 mg/mL for CS, 1,4-NQ and 1,2-NQ, respectively, while the MIC values against *S. epidermidis* were 0.323, 0.625 and 0.156 mg/mL for CS, 1,4-NQ and 1,2-NQ, respectively. Interestingly, 1,4-NQ exhibited higher antibacterial activity compared to CS and 1,2-NQ, being more effective against *S. epidermidis* after grafting. 

No improved antibacterial effects were noted for 1,2-NQ-CS. On the contrary, the grafting resulted in 2-fold or 4-fold MIC increases against *S. aureus* and *S. epidermidis*, respectively. Moreover, the antibacterial activity of 1,2-NQ-CS against *S. epidermidis* did not depend on the grafting method, with no differences in bacteria inactivation noted for the three applied methods. In contrast, 1,2-NQ-CS grafting by the EtOH:H_2_O method was essential in inhibiting *S. aureus* growth (Table 3). 

The superior antibacterial activity of 1,4-NQ against *S. aureus* was maintained in the presence of 1,4-NQ-CS, irrespective of grafting method. On the other hand, MIC values against *S. epidermidis* were 4-fold improved with the use of the EtOH:H_2_O and DMF methods, and 2-fold improved when employing the EtOH:H_2_O + Et_3_N method compared to 1,4-NQ (Table 3).

Considering that the CS modification using 1,4-NQ led to superior antimicrobial activities, these compounds were selected for further investigations as putative antitumoral compounds, testing them against human mammary adenocarcinoma cells.

### 3.10. Anticancer Potential of Naphthoquinone-Grafted Chitosan

**Figure 14:**
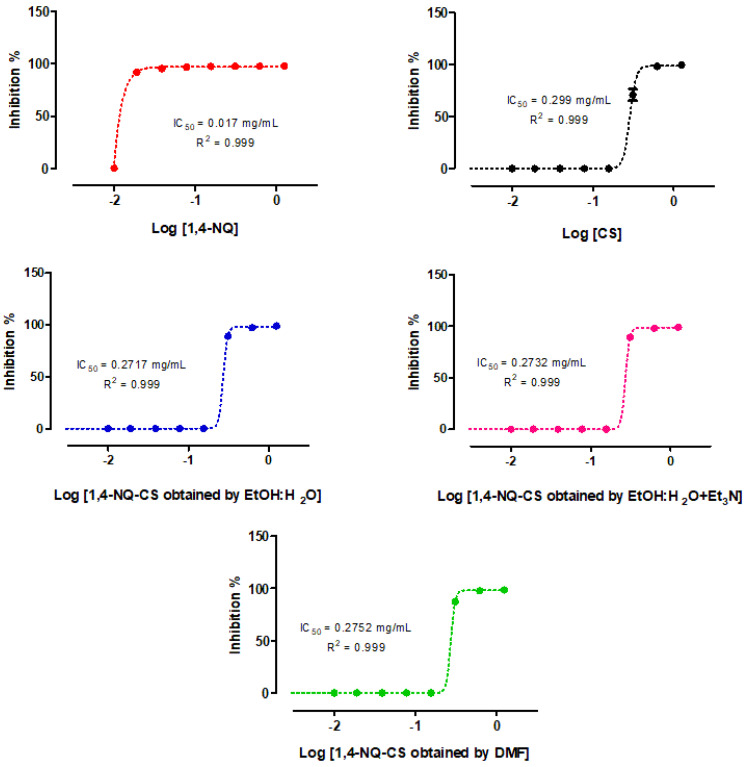
IC_50_ estimation from the proliferation curves of human mammary adenocarcinoma cells (MDA-MB-231) exposed for 24 h to increasing 1,4-naphthoquinone-grafted chitosan doses (1,4-NQ-CS) prepared by three different methods (EtOH:H_2_O, EtOH:H_2_O + Et_3_N, or DMF), unmodified chitosan (CS) and 1,4-naphthoquinone (1,4-NQ).

The proliferation of human mammary adenocarcinoma employing the MDA-MB-231 cell lineage was inhibited by 50% when exposed to CS and 1,4-NQ at 0.299 and 0.017 mg/mL, respectively, after 24 h of treatment (Figure 14). Independent of the employed CS modification methodology, the antitumoral effects of the products obtained by the three grafting methods were maintained at IC_50_ values of 0.27 mg/mL compared to CS when grafted with 1,4-NQ (Figure 14).

Altogether, these data indicate that the 1,4-NQ CS modifications conferred chemical stability and protection to the active compounds, also maintaining or improving their pharmacological properties and conferring final product biocompatibility.

### 3.11. Toxicological Evaluation against Healthy Human Cells and Therapeutic Index

The 50% cytotoxic concentrations (CC_50_) against healthy HFF-1 human fibroblasts were estimated from the proliferative curves in the presence of CS, 1,4-NQ and 1,4-NQ-CS after a 24 h exposure, producing a dose-dependent curve (Table 4). The high toxicity observed for 1,4-NQ at 0.027 mg/mL was mitigated after CS grafting, giving rise to compounds less toxic to healthy cells with 24–40-fold higher CC_50_ values (Table 4). 

The CC_50_/IC_50_ ratio, comprising the therapeutic index (TI), is a safety and effectiveness parameter, guaranteeing that the compound can be applied for a specific pharmacological purpose without producing toxic effects in healthy tissue. Regarding that therapeutic indices of 10 or above are widely considered as good, grafted CS was shown to be highly selective against *S. aureus* and *S. epidermidis*, except for 1,4-NQ-CS obtained by method c (DMF), which exhibited lower selectivity, with a TI under 10. However, in all antibacterial activity cases, CS modification with 1,4-NQ improved compounds safety and efficacy compared to CS and 1,4-NQ. The same was observed for MDA-MB-231, as a discrete increase in TI values for 1,4-NQ-CS was noted in comparison to CS and 1,4-NQ, although not achieving the adequate ratio of 10 or above (Table 4).

To decide or encourage new therapeutic approaches or drug repositioning involving antitumoral and/or antimicrobial compounds and its presentation, three parameters should be considered, namely toxicity to healthy cells, inhibition of tumoral cell proliferation, and the therapeutic index, or selective index, comprising a therapeutic product efficacy and safety parameters. Regarding TI values, CS grafted with 1,4-NQ by methods a and b are the best candidates for application as antimicrobials, with no risk of secondary effects if used at their effective dose.

*S. aureus* is the most prevalent bacterium associated with wounds, ulcers, subcutaneous abscesses, and skin infections, such as impetigo [54]. In contrast, *S. epidermidis* is a common human skin of that maintains skin homeostasis and protects against opportunistic pathogens, including *S. aureus*. However, *S. epidermidis* can also behave as an opportunist pathogen, found in skin diseases, implant-associated infections, nosocomial bloodstream infections and neonatal late-onset sepsis [55]. The use of a modified CS product, especially 1,4-NQ-CS, at the appropriate concentration could be useful in contributing to wound healing, protecting tissue against bacteria contamination until complete skin recovery. 

Regarding antitumoral activity, a discrete improvement in efficacy and safety from 1.58 to 2.98, 4.03 and 2.40 was noted comparing 1,4-NQ with its corresponding CS-grafted product. However, the therapeutic indices did not achieve the adequate level to ensure the absence of toxicity. Therefore, their application as an antitumoral agent should be considered with caution. 

Moreover, further studies are needed to investigate the molecular mechanisms of action activated by CS-grafted compounds, especially in controlling intracellular ROS levels, triggering bacteria and in MDA-MB-231 cell death or inhibition. Considering that reactive oxygen species (ROS) levels are crucial in the development of many diseases, including cancer, emerging medical formulations should have the ability or contain associated agents to regulate intracellular ROS levels, ensuring therapeutic efficacy. In this regard, the use of polymeric carriers associated with ROS-regulating molecules has been successfully demonstrated and deserves attention [56,57,58].

## 4. Conclusions

Chitosan presents functional groups, such as hydroxyl and amine groups, displaying a promising structure to prepare new derivatives with different bioactivities. In order to improve the antimicrobial properties associated with CS, in addition to investigating potential anticancer activity, two distinct naphthoquinones, sodium 1,2-naphthoquinone-4-sulfonate and the 2,3-dicholoro-1,4-naphthoquinone, were incorporated in the CS to produce new polymers. Three different reaction conditions were evaluated and all were proven efficient, confirmed by monitoring chemical structure changes by the analysis: FT-IR, TGA, DSC, CHN analysis, solid-state NMR and Raman spectroscopy. The antibacterial activity of the modified CS indicate that the 1,4-NQ-CS derivative exhibited higher safety and efficacy compared to CS and 1,4-NQ separately. Indeed, 1,4-NQ-CS antitumor tests indicated improved efficacy and safety, but their application as an antitumoral agent should be considered with caution, due to the threat of toxic effects. The results reported herein encourage further studies, especially concerning 1,4-NQ-CS, which may be useful against bacteria contamination. 

## Figures and Tables

**Table 1:** Elemental percentage (CHN), deacetylation degree (DD) and degree of substitution (DS) of chitosan (CS) and chitosan grafted with 1,2-naphthoquinone-4-sulfonate (1,2-NQ-CS) and with 1,4-naphtoquinone (1,4-NQ-CS) obtained by three different methods.

Sample	Method	C%	H%	N%	DD%	DS
**CS**	-	41.19	6.90	7.40	75.45	-
**1,4-NQ-CS**	EtOH:H_2_O	40.12	6.35	6.54	-	0.06
	EtOH:H_2_O + Et_3_N	41.24	6.32	6.12	-	0.12
	DMF	41.93	6.59	6.58	-	0.08
**1,2-NQ-CS**	EtOH:H_2_O	41.26	5.22	3.95	-	0.48
	EtOH:H_2_O + Et_3_N	47.61	4.84	4.33	-	0.54
	DMF	49.90	5.58	4.82	-	0.39

**Table 2:** Thermal properties of pure chitosan, 1,4-NQ-CS and 1,2-NQ-CS obtained by the three different methods (method a—EtOH:H_2_O; method b—EtOH:H_2_O + Et_3_N; and method c—DMF).

Sample	Method	T (ºC)	Weight Loss (%)	T (^o^C)	Weight Loss (%)
**CS**	-	93	7	304	38
**1,4-NQ-CS**	EtOH:H_2_O	67	3	268	32
	EtOH:H_2_O + Et_3_N	71	5	271	31
	DMF	73	5	273	24
**1,2-NQ-CS**	EtOH:H_2_O	78	10	263	20
	EtOH:H_2_O + Et_3_N	80	6	268	19
	DMF	82	6	262	25

**Table 3:** Minimum inhibitory concentration (MIC) of free-naphthoquinones, chitosan and chitosan grafted with different naphthoquinones against *Staphylococcus* of clinical interest.

Sample	Methods	Minimum Inhibitory Concentration (mg/mL)	
		*S. aureus* (ATCC 14458)	*S. epidermidis* (ATCC 12228)
CS	-	0.323	0.625
1,2-NQ	-	0.625	0.323
1,2-NQ-CS	EtOH:H_2_O	1.25	1.25
	EtOH:H_2_O + Et_3_N	NI	1.25
	DMF	NI	1.25
1,4-NQ	-	0.156	0.156
1,4-NQ-CS	EtOH:H_2_O	0.156	0.039
	EtOH:H_2_O + Et_3_N	0.156	0.078
	DMF	0.156	0.039

NI—no inhibition at the tested highest concentration. Minimum inhibitory concentrations were evaluated by the micro-dilution method in Mueller-Hinton broth in triplicate. *S. aureus* ATCC 14,458 and *S. epidermidis* ATCC 12,228 at 10^5^ CFU/mL were grown in the presence of increasing CS, 1,2-NQ, 1,4-NQ, 1,2-or NQ-CS concentrations obtained by the three methods.

**Table 4:** Cytotoxic concentrations (CC_50_) of 1,4-NQ-CS against a human healthy fibroblast lineage and therapeutic index (TI) calculation.

Estimated Indexes	Cell Lineage or Bacteria Lineage	CS	1,4-NQ	1,4-NQ-CS Method a	1,4-NQ-CS Method b	1,4-NQ-CS Method c
CC_50_ (mg/mL)	HFF-1 (HTB-26)	0.59	0.027	0.81	1.10	0.65
TI	MDA-MB-231 (SCRC-1041)	1.97	1.58	2.98	4.03	2.40
	*S. aureus* ATCC 14458	3.47	0.34	10.25 *	13.92 *	8.33
	*S. epidermidis ATCC 12228*	1.84	0.33	35.22 *	31.42 *	26.00 *

TI—Therapeutic index calculated as the CC_50_/IC_50_ ratio, where asterisk (*) indicates TI values over 10, indicating application safety. a, b, c—different methods for preparing 1,4-NQ-grafted CS. CS—chitosan; 1,4-NQ—1,4 naphthoquinone; 1,4-NQ-CS—chitosan grafted with 1,4 naphthoquinone by method a (EtOH:H_2_O), method b (EtOH:H_2_O + Et_3_N), and method c (DMF).

## Data Availability

Data supporting reported results can be found in the manuscript.

## Supplementary Materials

The following supporting information can be downloaded at: https://www.mdpi.com/article/10.3390/polym15061430/s1, Figures S1–S9: FTIR spectra of samples CS, 1,2-NQ, 1,2-NQ-CS (A-C methods), 1,4-NQ and 1,4-NQ-CS (A-C methods); Figures S10–S18: ^13^C NMR solid state spectra of CS, 1,2-NQ, 1,2-NQ-CS (A-C methods), 1,4-NQ and 1,4-NQ-CS (A-C methods).
