# Supplementary material for: Exploring the Antimicrobial and Antitumoral Activities of Naphthoquinone-Grafted Chitosans
Source: Polymers (Basel). 2023 Mar 14;15(6):1430. doi: 10.3390/polym15061430 (PMC10053705; doi:10.3390/polym15061430)
Supplement: Supplementary file 1 [file polymers-15-01430-s001.zip › polymers-2180084-supplementary.pdf]

# Supplementary Materials

**Figure S1-S9.** FTIR spectra of samples CS, 1,2-NQ, 1,2-NQ-CS (A-C methods), 1,4-NQ and 1,4-NQ-CS (A-C methods).

**Figure S10-S18.** $^{13}C$ NMR solid state spectra of CS, 1,2-NQ, 1,2-NQ-CS (A-C methods), 1,4-NQ and 1,4-NQ-CS (A-C methods).

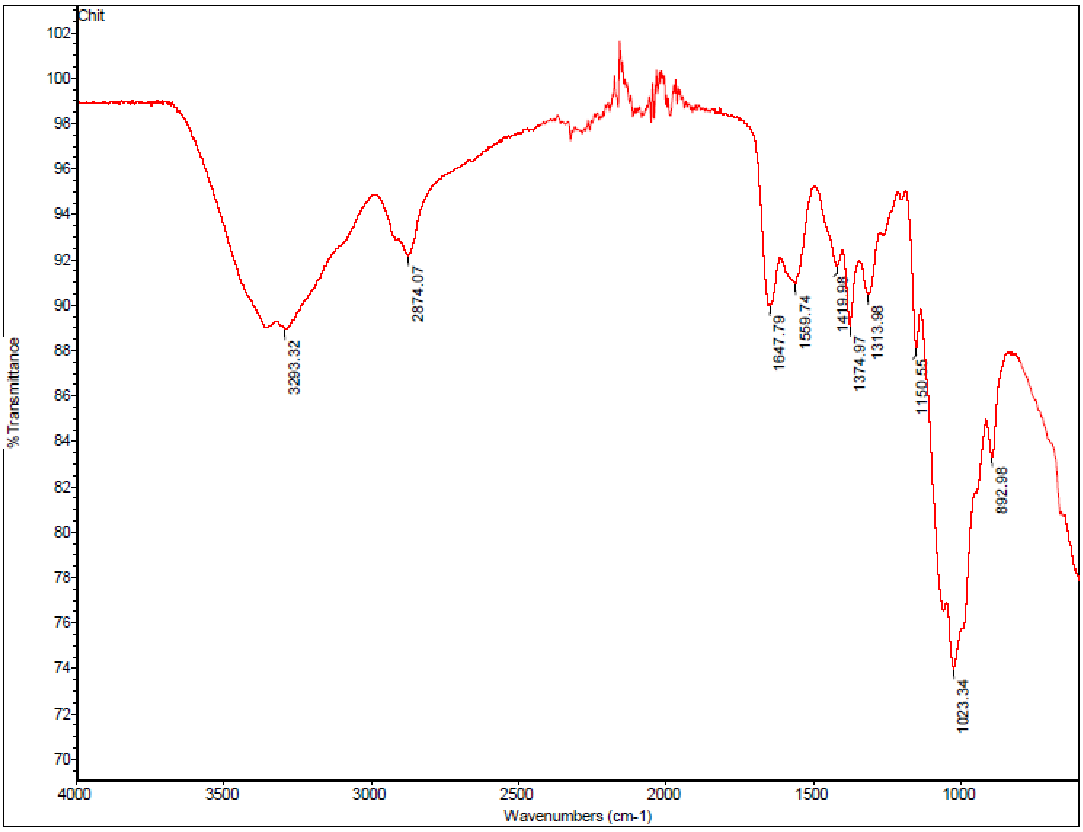


**Figure S1.** FTIR spectra of pure chitosan.

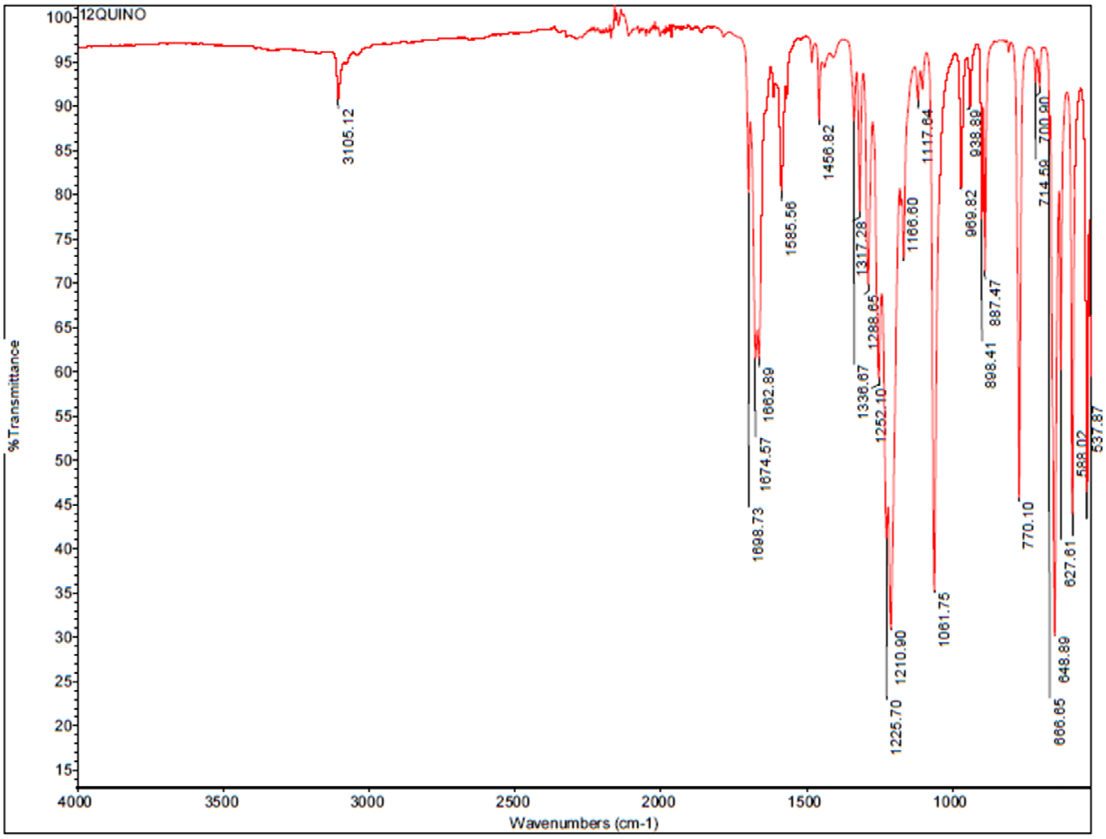


**Figure S2.** FTIR spectra of pure 1,2-NQ.

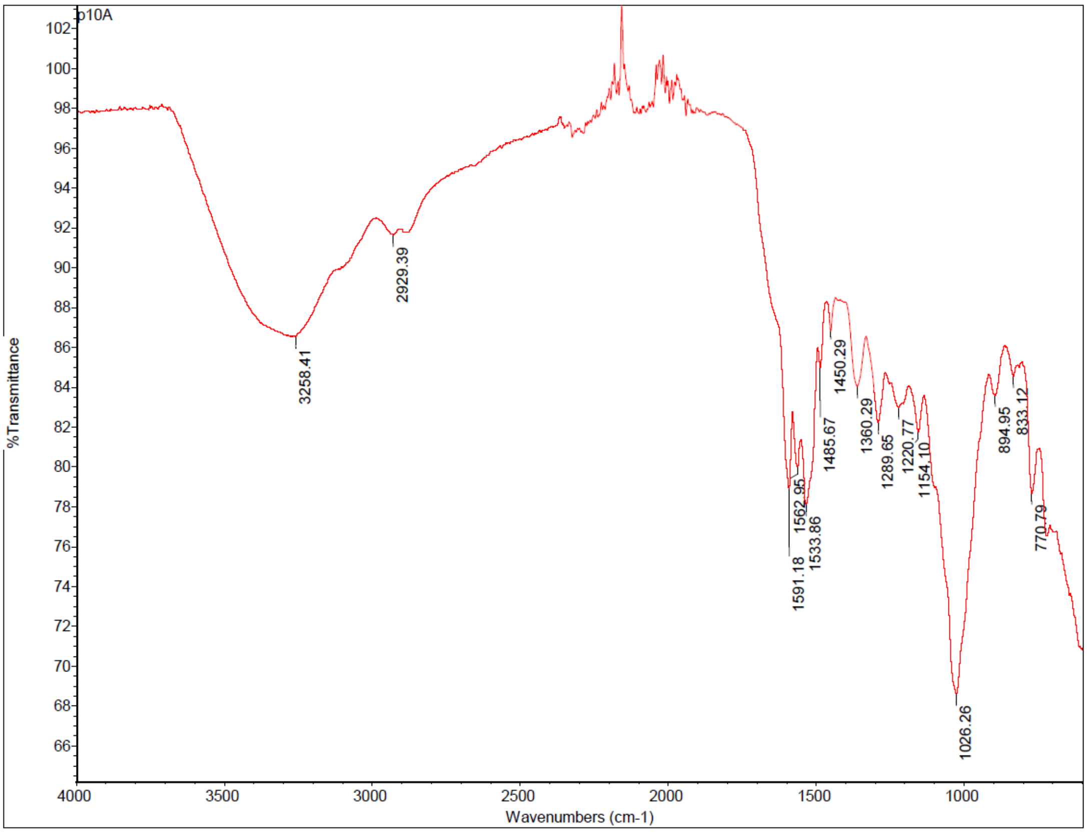


**Figure S3.** FTIR spectra of 1,2-NQ-CS obtained by method a.

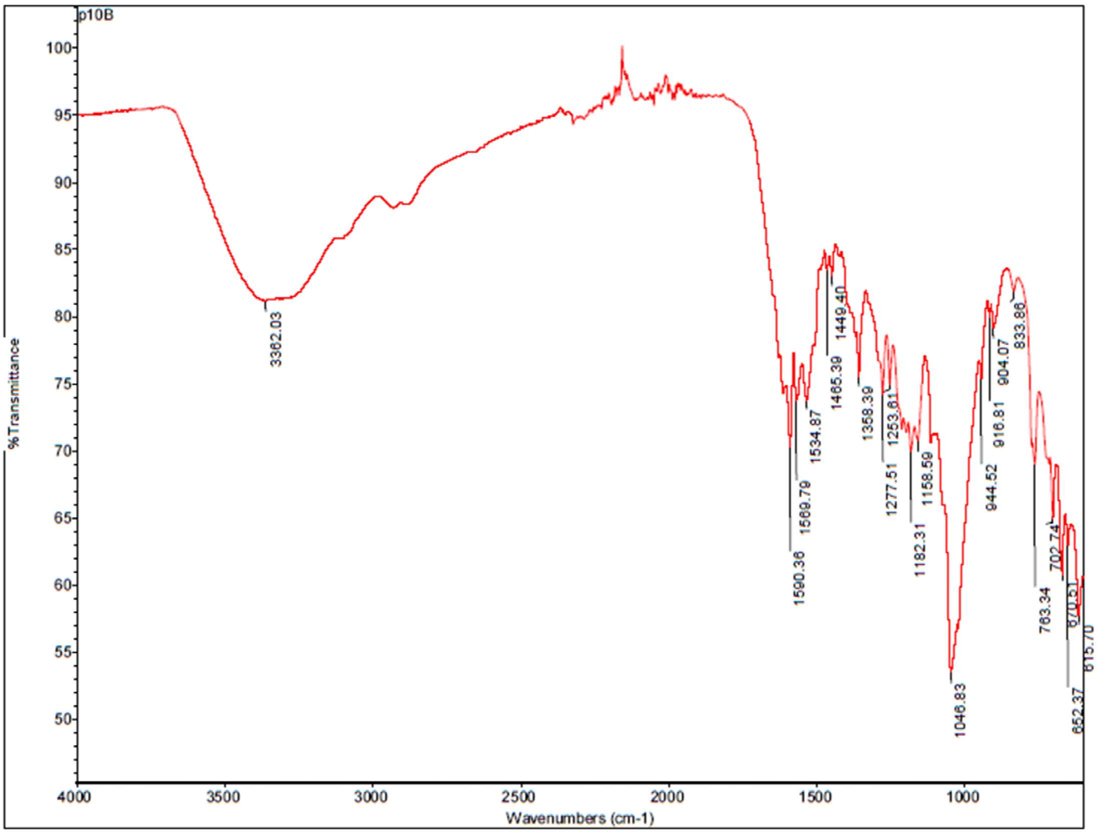


**Figure S4.** FTIR spectra of 1,2-NQ-CS obtained by method b.

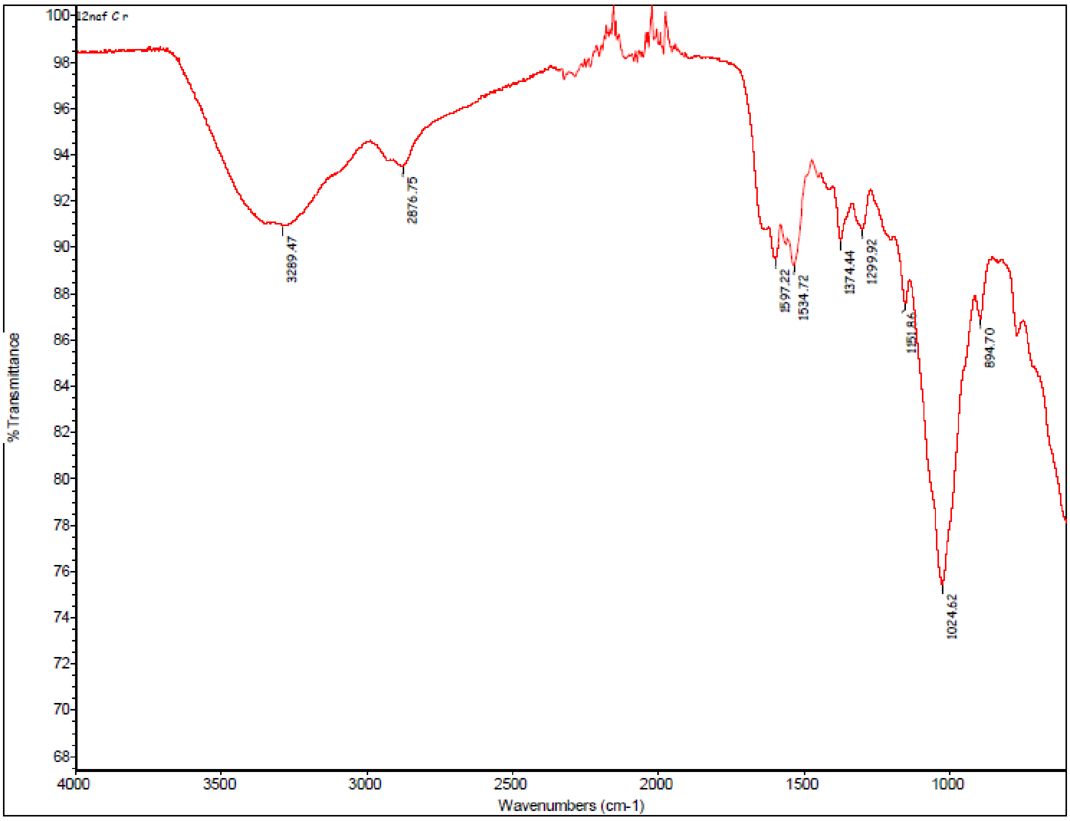


**Figure S5.** FTIR spectra of 1,2-NQ-CS obtained by method c.

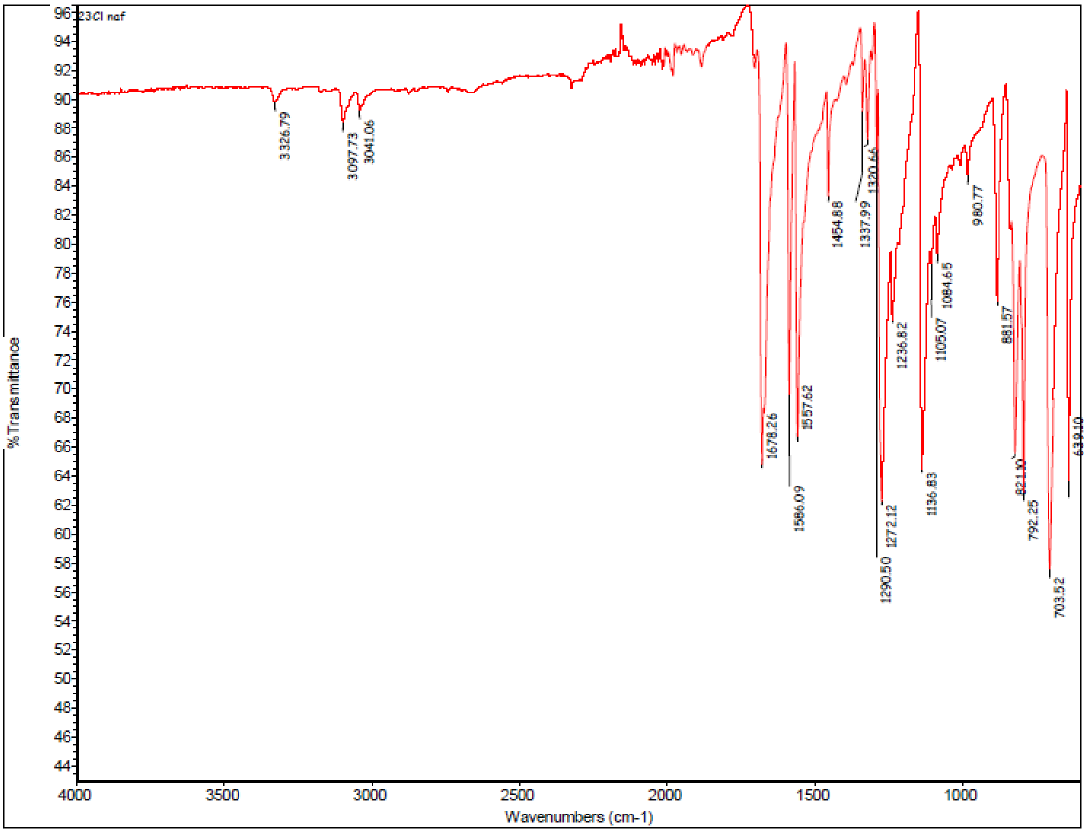


**Figure S6.** FTIR spectra of pure 1,4-NQ.

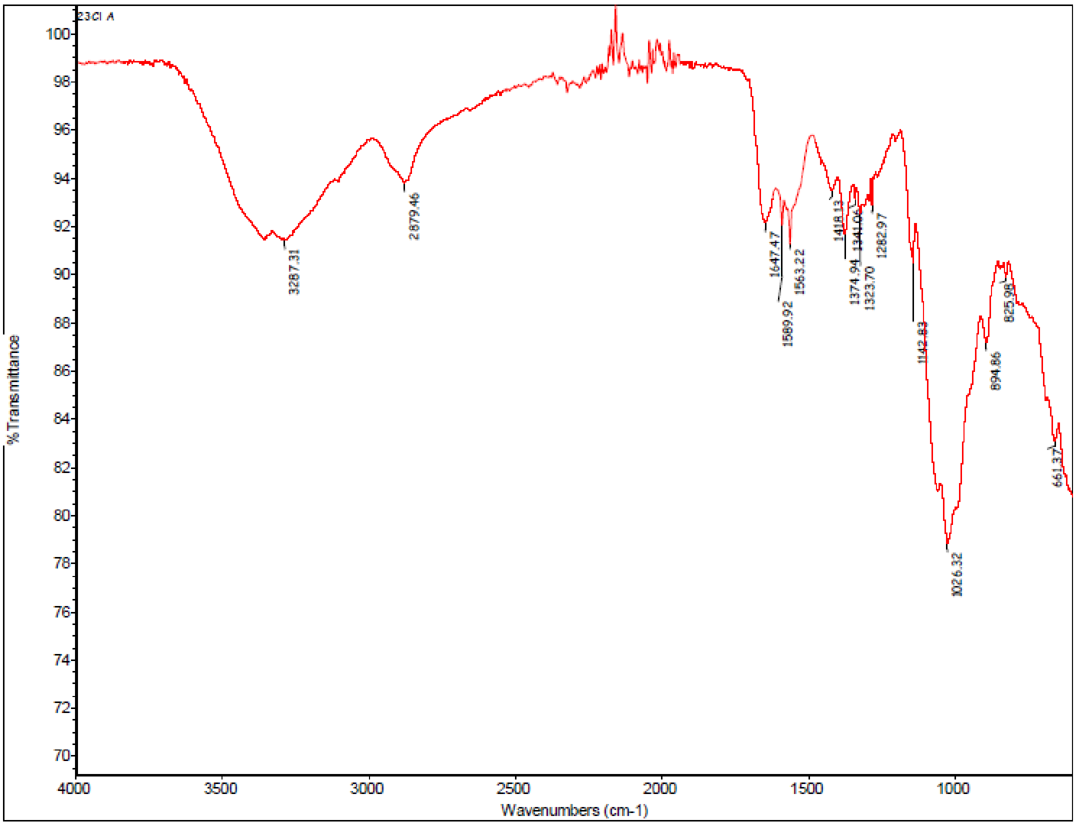


**Figure S7.** FTIR spectra of 1,4-NQ-CS obtained by method a.

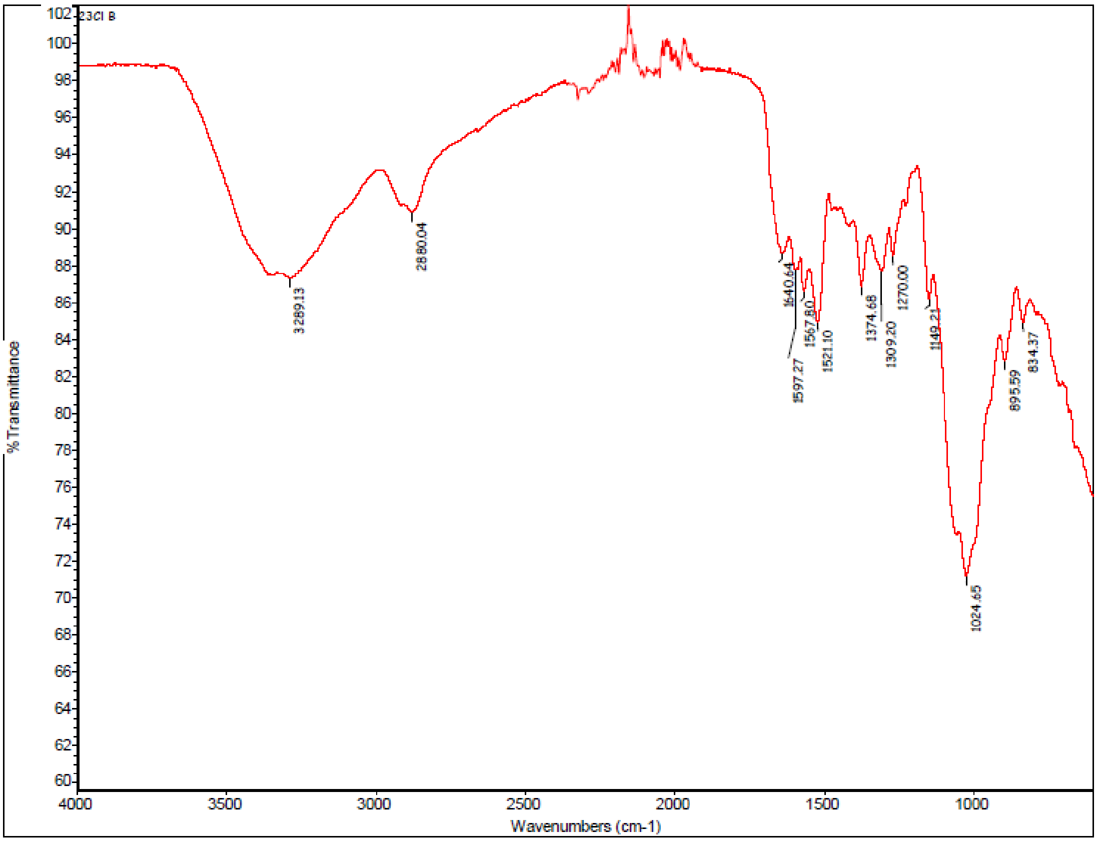


**Figure S8.** FTIR spectra of 1,4-NQ-CS obtained by method b.

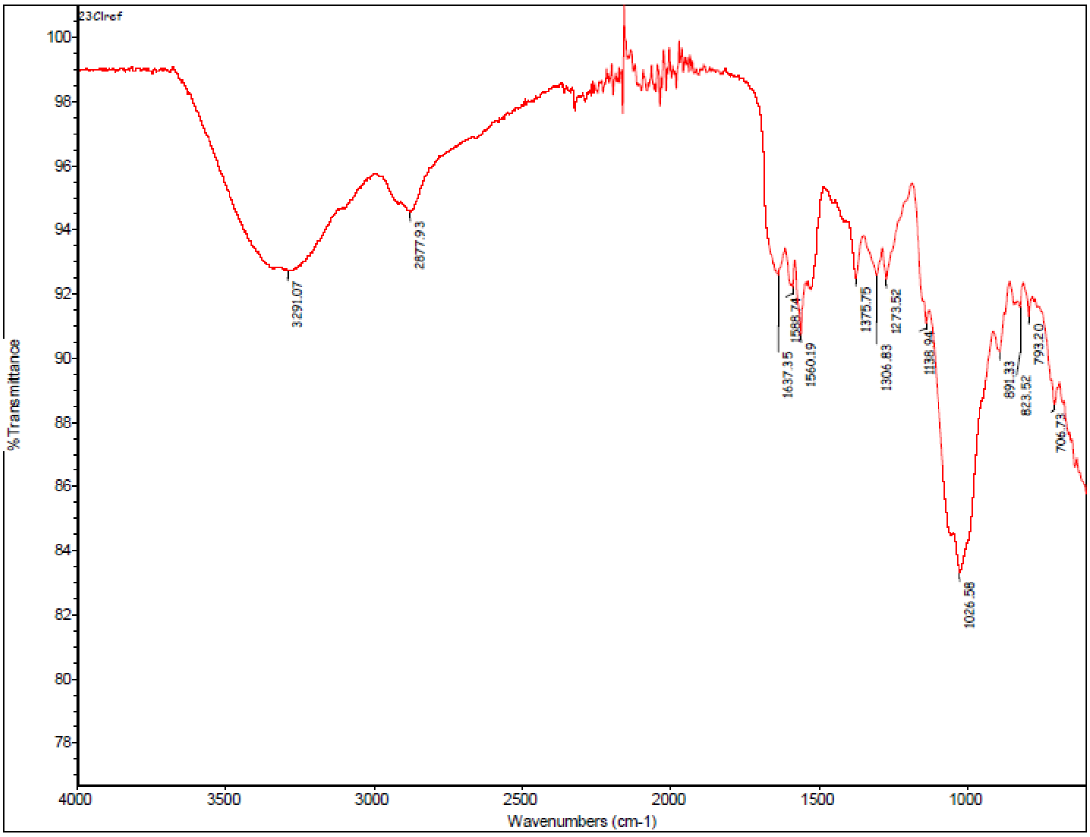


**Figure S9.** FTIR spectra of 1,4-NQ-CS obtained by method c.

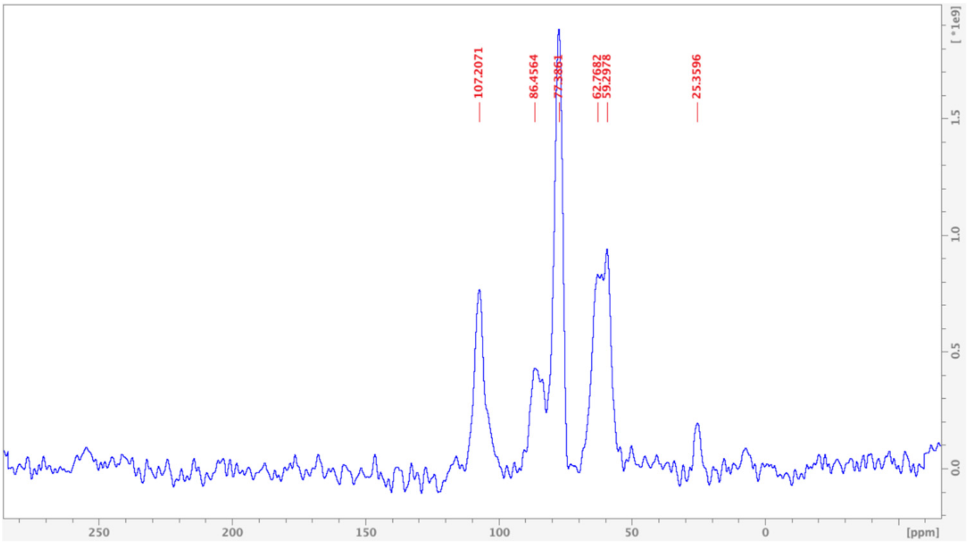


**Figure S10.** 125 MHz of $^{13}C$ Solid State CP/MAS-10 KHz NMR spectra of CS.

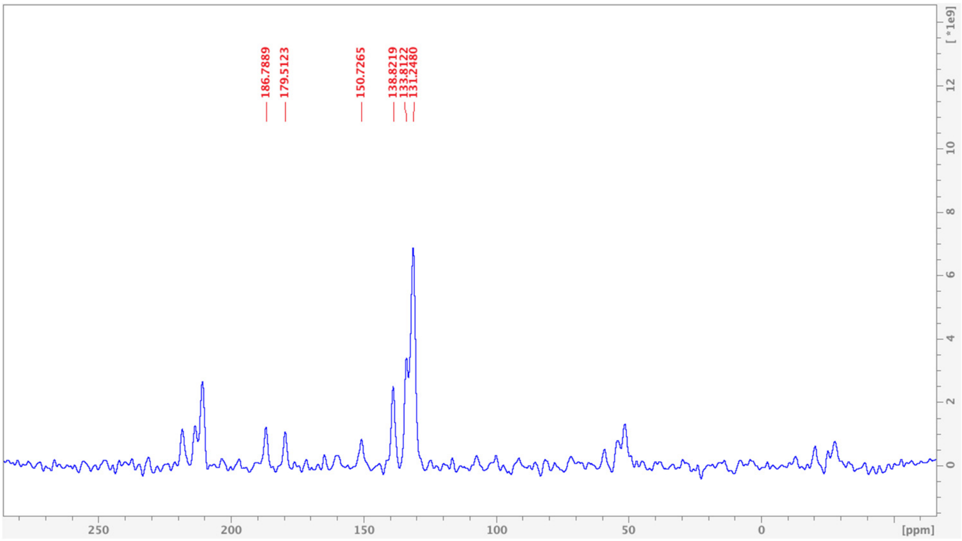


**Figure S11.** 125 MHz of $^{13}C$ Solid State CP/MAS-10 KHz NMR spectra of 1,2NQ.

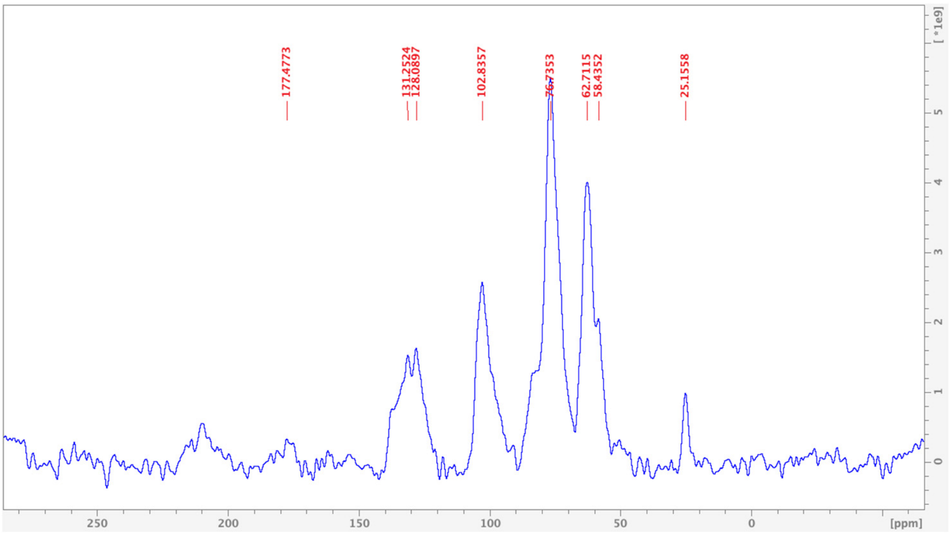


**Figure S12.** 125 MHz of $^{13}C$ Solid State CP/MAS-10 KHz NMR spectra of 1,2NQ-CS obtained by method a.

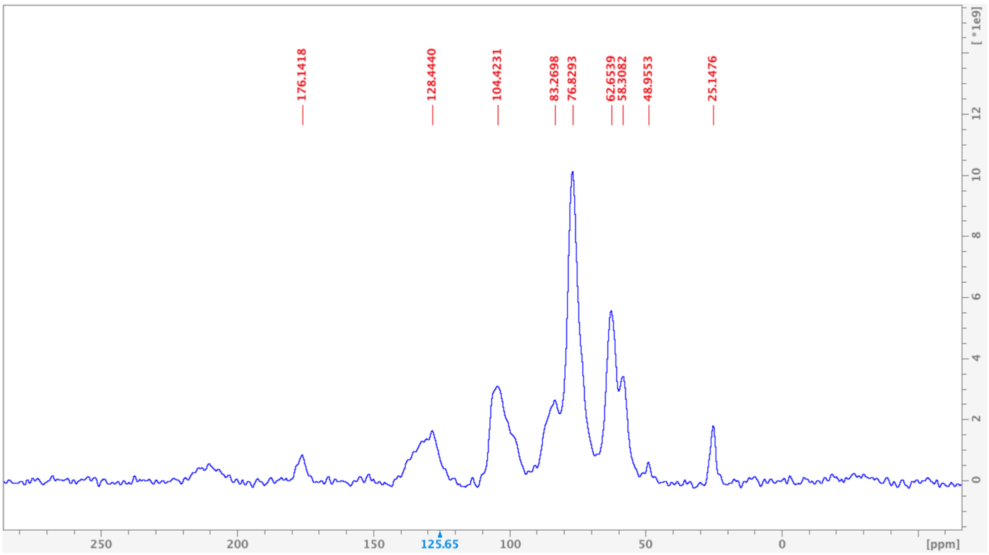


**Figure S13.** 125 MHz of $^{13}C$ Solid State CP/MAS-10 KHz NMR spectra of 1,2NQ-CS obtained by method b.

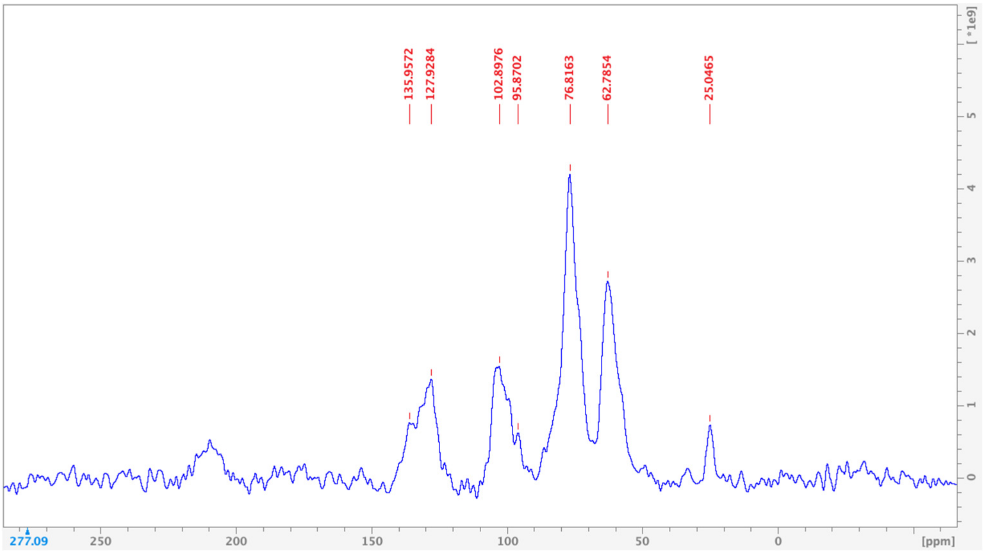


**Figure S14.** 125 MHz of $^{13}C$ Solid State CP/MAS-10 KHz NMR spectra of 1,2NQ-CS obtained by method c.

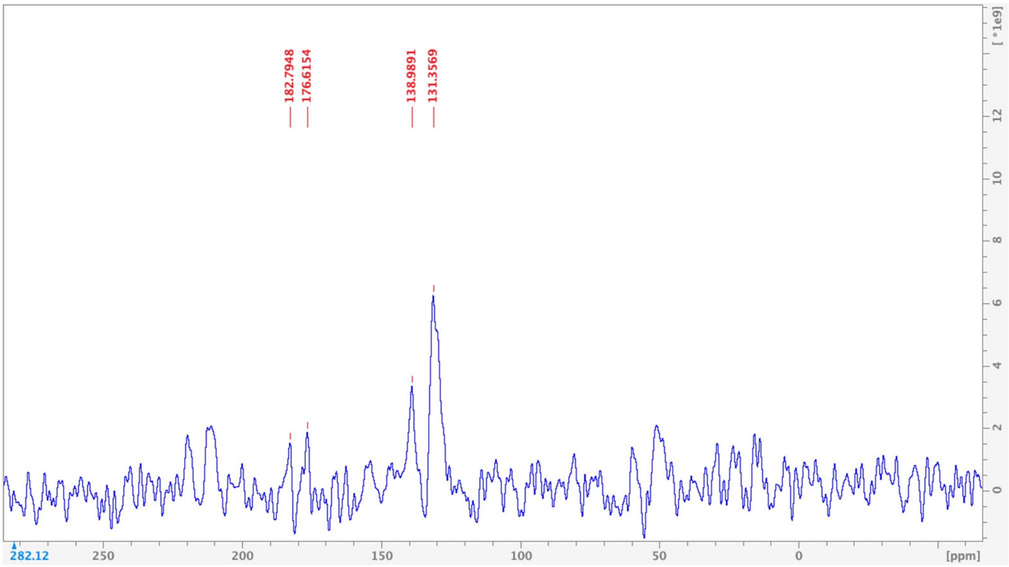

**Figure S15.** 125 MHz of $^{13}C$ Solid State CP/MAS-10 KHz NMR spectra of 1,4-NQ.

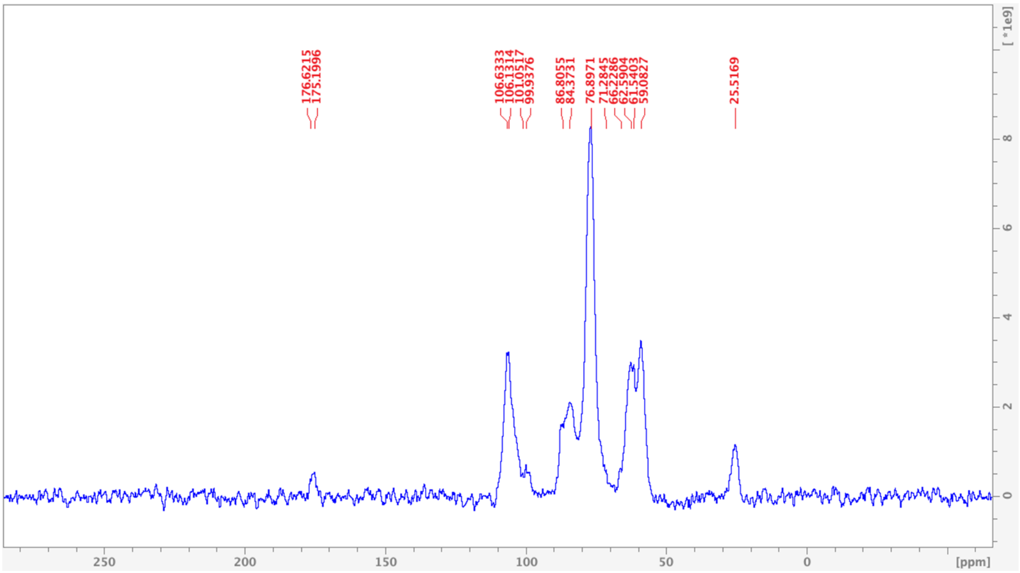


**Figure S16.** 125 MHz of $^{13}C$ Solid State CP/MAS-10 KHz NMR spectra of 1,4-NQ-CS obtained by method a.

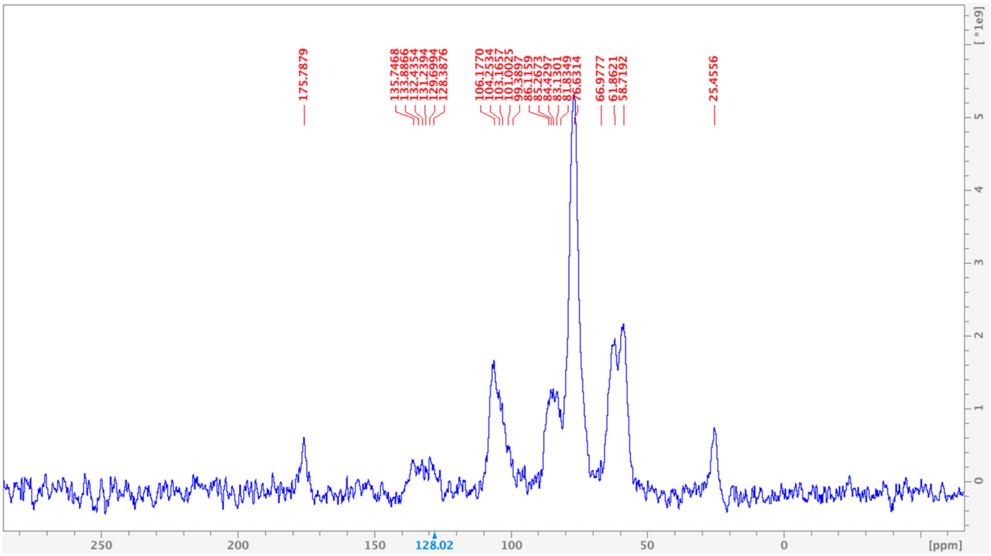


**Figure S17.** 125 MHz of $^{13}C$ Solid State CP/MAS-10 KHz NMR spectra of 1,4-NQ-CS obtained by method b.

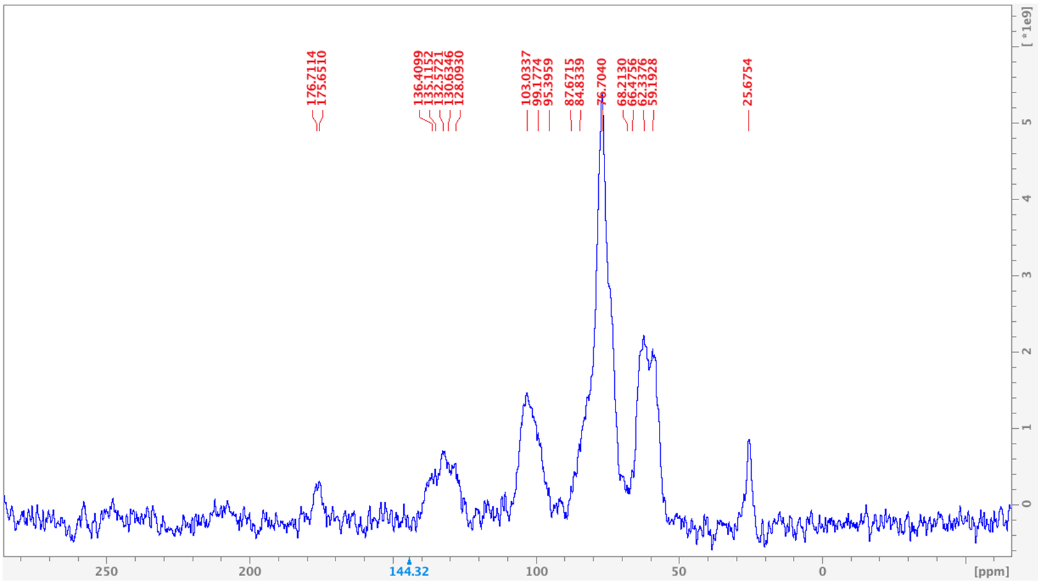


**Figure S18.** 125 MHz of $^{13}C$ Solid State CP/MAS-10 KHz NMR spectra of 1,4-NQ-CS obtained by method c.
